# A protective and heterosubtypic antibody lineage targeting the influenza A virus neuraminidase active site

**DOI:** 10.1172/JCI188887

**Published:** 2025-12-01

**Authors:** Ty A. Sornberger, Rachael M. Wolters, Iuliia M. Gilchuk, Luke Myers, Elad Binshtein, Ryan Irving, Elaine C. Chen, Pavlo Gilchuk, Rachel S. Nargi, Rachel E. Sutton, Bethany N. Howard, Laura S. Handal, Andrew Trivette, Katherine E. Webb, Chandrahaas Kona, Eduardo Villalobos, Lauren E. Williamson, James E. Crowe, Seth J. Zost

**Affiliations:** 1 Department of Pathology, Microbiology, and Immunology and; 2 Vanderbilt Center for Antibody Therapeutics, Vanderbilt University Medical Center, Nashville, Tennessee, USA.; 3 Department of Biomedical Engineering, Vanderbilt University, Nashville, Tennessee, USA.; 4 Department of Pediatrics, Vanderbilt University Medical Center, Nashville, Tennessee, USA.

**Keywords:** Immunology, Infectious disease, Adaptive immunity, Immunoglobulins, Influenza

## Abstract

Influenza type A viruses (IAVs) remain an extraordinary burden to global public health and regularly circulate through human populations. This investigation describes the isolation of human mAbs from an individual with a substantial history of influenza exposure via vaccination and natural infection. From these mAbs, a clonally expanded B cell lineage was identified that recognizes the IAV neuraminidase (NA) glycoprotein and binds near the NA active site of H3N2 viruses to inhibit sialidase activity. Further characterization found that some somatically mutated members of this lineage exhibited cross-reactive binding to recombinant N1 and N9 antigens, suggesting that heterosubtypic reactivity was acquired through somatic mutation. Two candidate mAbs from this family — FluA-168 and FluA-173 — potently inhibited IAV replication in vitro and protected against lethality in vivo. The results of this study contribute to our understanding of cross-reactivity between IAV subtypes in response to diverse exposure patterns and identified 2 mAbs as potential therapeutic candidates for IAV infection.

## Abstract 2

An antibody lineage from a single donor binds the active site of influenza neuraminidase, cross-reacts with antigenically diverse viruses, and protects mice from infection.

## Introduction

Influenza is a viral respiratory disease that affects an estimated 1 billion people and results in more than 500,000 deaths annually (1, 2). Prophylactic vaccines and antiviral therapies can provide protective immunity by targeting the viral glycoproteins HA, neuraminidase (NA), and matrix-2 (M2) to inhibit major functions in the virus replication cycle (3–5). The major role of NA in the influenza virus replication cycle is the enzymatic cleavage of sialic acid residues at the surface of infected respiratory epithelium cells, allowing newly formed virions to egress and infect other susceptible cells (6–12). In addition, other NA mechanisms of action have been proposed, including migration of virions through the respiratory mucosa by cleaving mucins (13, 14), indirect enhancement of HA receptor-binding activity (15), and cooperative binding of sialic acid via NA receptor-binding sites (16–23). Small-molecule NA inhibitors (NAIs), such as oseltamivir and zanamivir, have been used to inhibit NA activity during acute infections; however, influenza viruses are becoming resistant to these molecules due to mutations in the NA active site and require use within 48 hours of symptom onset (24–28). The limitations of small-molecule NAIs necessitate the discovery and development of alternative therapeutic options for influenza infection, such as mAbs, which have been shown to effectively reduce respiratory viral load and mortality in influenza patients (29). Antibodies that target conserved epitopes of the NA active site and inhibit its enzymatic function demonstrate neutralization activity across multiple IAV subtypes and are correlates of protection for in vivo efficacy (30–32).

The effectiveness of long-lasting NAI mAbs, however, is complicated by the constant evolution of influenza viruses. Due to the inability of the viral RNA polymerase to proofread during replication, viral mRNA and genomic transcripts frequently contain errors, resulting in an accumulation of point mutations in viral antigens referred to as “antigenic drift.” The HA and NA surface glycoproteins are largely amenable to these mutations, provided they do not dramatically affect viral fitness, and often confer increased transmissibility, cell tropism, or resistance to small molecules or neutralizing antibodies elicited from previous exposures (33–41). This process is a driving force for seasonal epidemics and contributes to the public health burden imposed by influenza, as this antigenic drift necessitates the reformulation of annual vaccines.

While NA can mutate and acquire NAI resistance, previous studies have suggested that NA is more conserved than HA and does not mutate as frequently, making it an attractive target for therapeutic development (42, 43). Human influenza challenge studies have also demonstrated that high titers of NAI antibodies are associated with protection and, as shown more recently, are an independent correlate of protection from hemagglutination-inhibition titers (44–46).

Additionally, studies show that naturally infected individuals produce heterogeneous humoral responses to HA, NA, and other viral proteins, whereas vaccination heavily skews toward the induction of HA-reactive antibodies (3, 12, 47, 48). Together, these data suggest that NA-directed humoral immunity also plays an important role in the prevention and clearance of influenza virus infection.

Here, we describe the expansion of a clonal B cell lineage recognizing the NA of H1N1, H3N2, and H7N9 influenza type A virus (IAV) subtypes amid circulating plasmablasts or memory B cells of an individual who had been vaccinated with diverse licensed and experimental influenza vaccines and naturally infected. Individual cells were sequenced for paired heavy- and light-chain antibody sequences, and the encoded antibodies were expressed recombinantly to generate a panel of 22 clonally related mAbs. We also synthesized and expressed the computationally inferred unmutated common ancestors (UCAs) that represent the genetic origin of the lineage. Longitudinal analysis of this lineage indicated that heterosubtypic binding was not germline encoded and likely arose from somatic mutations introduced during affinity maturation, as memory B cells were recruited in response to sequential antigen exposures. Of this panel, we identified 2 somatically mutated mAbs, designated FluA-168 and FluA-173, that exhibited broad binding to H1N1, H3N2, and H7N9 antigens; potently inhibited sialidase activity through direct binding to the NA active site; and protected mice from mortality in a virus challenge model following passive immunization with mAb. Thus, this study identified a clonal B cell lineage that acquired cross-reactivity to 3 IAV NA subtypes and potently inhibited NA sialidase activity. Together, these findings suggest that exposure to diverse NA antigens via vaccination and natural infection induces a cross-reactive B cell response encoding beneficial antibodies that inhibit IAV NA enzymatic activity.

## Results

### Isolation and characterization of a B cell lineage reactive to N1, N2, and N9 IAV NA subtypes.

**Figure 1:**
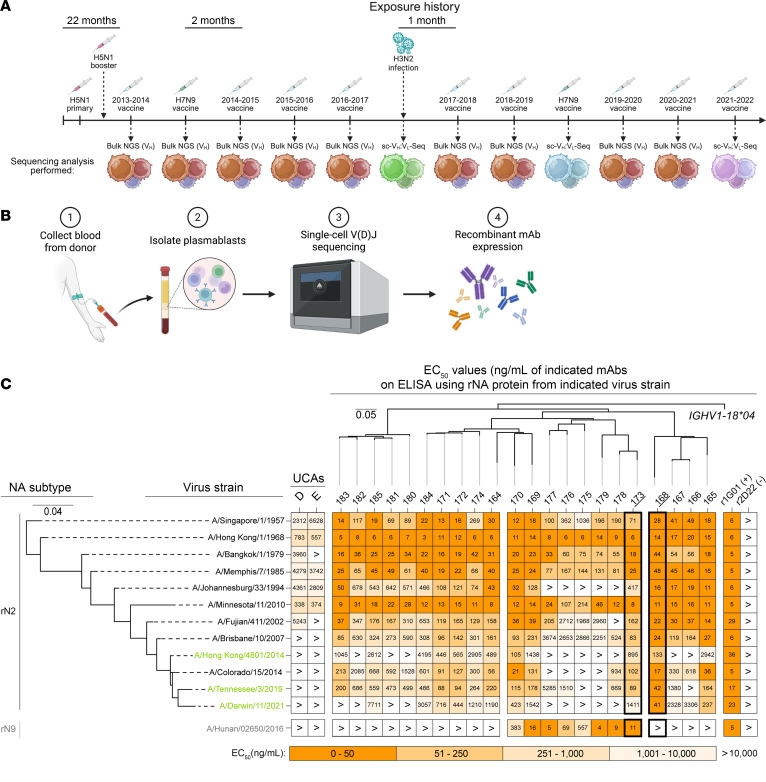
Identification and characterization of a FluA B cell lineage. (**A**) Timeline illustrating the exposure history of the donor with types of exposures and sequencing indicated. (**B**) Illustration of the general antibody discovery process in which plasmablasts are isolated and sequenced using single-cell sequencing techniques, and the antibodies are recombinantly expressed. Illustrations in **A** and **B** created in BioRender. (**C**) ELISA heatmap indicating mAb binding potency to rN2 and rN9. Columns represent mAbs used, and rows represent the indicated IAV strains. Phylogenetic trees indicate the divergence of (top) full-length heavy-chain nucleotide sequences of the FluA B cell lineage from the germline heavy-chain variable gene or (left) the relatedness of the NA nucleotide sequence between each IAV strain shown in the heatmap over time. Scale bars: average number of nucleotide substitutions between each node. Binding potency is represented as an EC_50_ value. Black indicates IAV N2 strains without an N-linked glycan motif at position 245 of NA (S245/S247), and green indicates IAV N2 strains containing an N-linked glycan motif at position 245 of NA (N245/T247). Candidate mAbs FluA-168 and FluA-173 are highlighted. All mAbs were assessed in triplicate, and data are representative of 2 biological replicates.

Peripheral blood was collected annually from a healthy male individual (designated as “donor 269”) with an extensive history of seasonal influenza vaccinations, including an initial H5N1 vaccination (A/Vietnam/1203/2004) and booster (A/Indonesia/05/2005) and 2 H7N9 vaccinations (A/Shanghai/2/2013 and A/Hong Kong/125/2017) as part of NIH-sponsored vaccine clinical trials (Figure 1A and Supplemental Figure 1; supplemental material available online with this article; https://doi.org/10.1172/JCI188887DS1). In 2017, we isolated plasmablasts from this individual 7 days after the onset of clinical symptoms associated with an acute, laboratory-confirmed H3N2 IAV infection. We recovered more than 2,000 paired heavy- and light-chain variable gene sequences from this cell population using a commercial single-cell sequencing platform (10x Genomics) (49). Plasmablasts were also isolated and sequenced in 2019 and 2021, each 7 days after immunization, with an experimental H7N9 vaccine and a licensed 2021–2022 quadrivalent seasonal influenza vaccine, respectively (Supplemental Figures 1–3). We recombinantly expressed these mAbs using high-throughput microscale expression, as previously described (50), and screened for reactivity using ELISA to several IAV antigens, including recombinantly expressed NA (rNA) proteins (Figure 1B). Of the antibody clones tested, 2 clonally related mAbs — later named FluA-169 and FluA-170 — bound to both rN2 (A/Brisbane/10/2007) and rN9 (A/Hunan/2650/2016) antigens. This lineage, designated as mAbs numbered FluA-164 through FluA-185, is encoded by the antibody genes IGHV1-18*04 and IGKV1-5*01. Clustering of this lineage with single-cell sequencing data from donor 269 identified 22 unique sequences.

### Evolutionary analysis identifies a spectrum of cross-reactivity to N2 and N9 antigens.

We used phylogenetic inference methods to identify the UCA sequence of these 22 antibodies (i.e., the sequence that likely represents the ancestral naive B cell receptor (BCR) of the FluA B cell lineage prior to antigen exposure). From this, we inferred 2 possible UCAs that encode either an aspartic acid (D) or glutamic acid (E) residue at position 108 in heavy-chain complementarity-determining region 3 (HCDR3); these are denoted as UCA D and UCA E, respectively. We recombinantly expressed all 22 mAbs and their UCAs at midi-scale (~1 mg) and screened for binding to a panel of rN2 antigens corresponding to major H2N2 or H3N2 IAV strains circulating during the years 1957–2021. We also included an rN9 antigen derived from the H7N9 strain A/Hunan/2650/2016 to assess cross-reactivity. As a positive control, we included a recombinant version of the previously described mAb 1G01 (r1G01) that binds to the NA active site of all IAV subtypes and both IBV lineages (30). We also included a recombinant version of a previously described dengue virus–reactive mAb (rDENV-2D22, or r2D22) as an isotype-matched negative control (51). Based on these results, the inferred UCAs reacted to most of the rN2 antigens from 1957–1994; however, binding to the rN9 antigen was not observed. These results suggest that the heterosubtypic binding pattern for N2 and N9 subtypes exhibited by some members of this clonal family may not be germline-encoded (Figure 1C).

During the 2014–2015 influenza season, circulating H3N2 IAV strains acquired 2 amino acid substitutions (S245N/S247T) within NA, introducing an N-linked glycan motif at position 245. This glycan shields the NA active site, preventing binding and inhibition by most anti-NA antibodies (52). As expected, many mAbs from this lineage showed considerably decreased levels of binding to the A/Hong Kong/4801/2014 strain, which contains the glycan. Genetically removing the glycan at position 245 from A/Hong Kong/4801/2014 NA (sequence-matched to A/Colorado/15/2014) improved the binding reactivity of 18 of the 22 somatically mutated mAbs tested (Figure 1C). This finding indicates that mAbs of this clonal lineage likely bind at or near the active site of N2. Additionally, the binding of r1G01 did not substantially decrease when tested against the A/Hong Kong/4801/2014 rN2 antigen (30), suggesting that mAbs of this lineage may differ from r1G01 in their angle of approach to the N2 active site.

### H3N2 and H7N9 NA sialidase activity is inhibited by NA-reactive mAbs.

**Figure 2:**
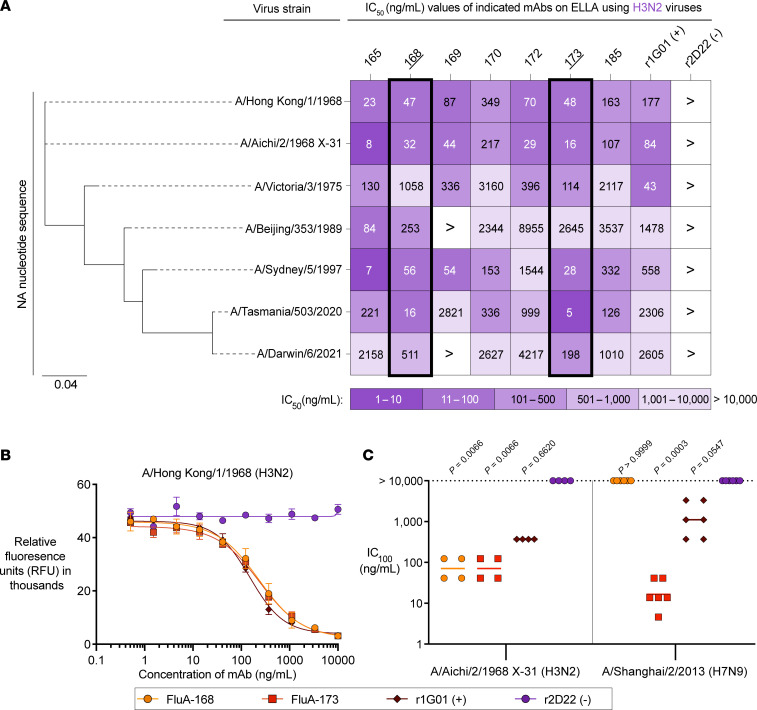
mAbs inhibit sialidase activity and prevent viral egress. (**A**) ELLA heatmap indicating the potency of mAb-mediated inhibition of NA sialidase activity measured on a panel of H3N2 viruses. Columns represent mAbs used, and rows represent the indicated H3N2 virus strains. The potency of NA sialidase activity inhibition is represented as a half-maximal inhibitory concentration (IC_50_) value. Candidate mAbs FluA-168 and FluA-173 are highlighted. The phylogenetic tree indicates the relatedness of the NA nucleotide sequence between each IAV strain. Scale bar: the average number of nucleotide substitutions between the indicated virus strains. All mAbs were assessed in triplicate across 2 biological replicates. (**B**) MUNANA-based assay showing NA sialidase inhibition of an H3N2 virus (A/Hong Kong/1/1968) by candidate mAbs FluA-168 and FluA-173, positive control mAb r1G01, and isotype-negative control mAb r2D22. Data points and error bars indicate mean ± SD. All mAbs were assessed in triplicate across 3 biological replicates. (**C**) Egress inhibition assay of an (left) H3N2 virus (A/Aichi/2/1968 X-31) and (right) H7N9 virus (A/Shanghai/2/2013-PR8-IDCDC-RG32A) showing potency of candidate mAbs FluA-168 and FluA-173, positive control mAb r1G01, and isotype-negative control mAb r2D22. Data points indicate IC_100_ values, with the geometric mean shown. All mAbs were assessed in (left) duplicate or (right) triplicate across 2 biological replicates. Adjusted *P* values were calculated using the Kruskal-Wallis test with Dunn’s multiple-comparison post hoc test. The statistical significance threshold set to α ≤ 0.05.

An enzyme-linked lectin assay (ELLA) was used to assess inhibition of NA enzymatic activity by candidate mAbs of this B cell lineage. The ELLA uses a large-molecule fetuin glycoprotein containing N- and O-linked glycans with terminal sialic acid residues. Cleavage of these sialic acids by NA exposes the penultimate galactose moieties, which are then recognized by the lectin peanut-agglutinin-horseradish peroxidase (PNA-HRP). Inhibition of NA sialidase activity by mAbs can be measured dose-dependently based on the signal produced by HRP (10). Of the panel of 22 somatically mutated mAbs screened by ELISA, 7 mAbs with potent binding reactivity to rNA antigen were chosen for functional characterization by ELLA against 7 H3N2 viruses representing circulating strains from 1968 to 2021. Two mAbs — FluA-168 and FluA-173 — demonstrated potent inhibition of NA enzymatic activity against the most modern H3N2 virus strain, A/Darwin/6/2021 (Figure 2A).

The functionality of FluA-168 and FluA-173 was further characterized using both an NA-Fluor and an egress inhibition assay. While the ELLA uses a large-molecule substrate to mimic realistic interactions between mAbs and NA, inhibition of NA sialidase activity may be due to steric hindrance rather than direct binding to the NA active site. The NA-Fluor assay uses a small-molecule substrate, 2′-(4-methylumbelliferyl)-α-d-N-acetylneuraminic acid (MUNANA), that resembles sialic acid and fluoresces upon cleavage. Due to the much smaller size of the MUNANA substrate, cleavage necessitates that a mAb bind directly to or allosterically inhibit the NA active site (3, 10, 30, 53). Candidate mAbs were tested against an H3N2 virus (A/Hong Kong/1/1968) using this MUNANA-based assay. FluA-168, FluA-173, and r1G01 inhibited NA sialidase activity compared with the isotype-negative control, r2D22 (Figure 2B, Supplemental Figure 11). These data suggest that both mAbs directly inhibit H3N2 NA sialidase activity by binding to the active site and may reflect their functionality against other IAV virus subtypes.

Cleavage of sialic acids decorating host cell proteins is required for efficient egress of nascent virions from the cell surface (54). To assess the ability of FluA-168 and FluA-173 to inhibit viral egress, we used a previously described viral egress inhibition assay that assesses hemagglutination activity by cell supernatants as an indicator for the presence of viral particles (55). We tested the ability of FluA-168 and FluA-173 to inhibit egress of the H3N2 strain A/Aichi/2/1968 X-31 and a recombinant H7N9 virus expressing the H7 and N9 genes of A/Shanghai/2/2013. FluA-168, FluA-173, and r1G01 all inhibited the egress of A/Aichi/2/1968 X-31 virions from cells, with mean IC_100_ values of 71.3, 71.3, and 370.4 ng/mL, respectively (Figure 2C). However, only FluA-173 and r1G01 inhibited the egress of A/Shanghai/2/2013 virions from cells, with mean IC_100_ values of 16.5 and 1,111.1 ng/mL, respectively (Figure 2C). Together, the results of the ELLA, MUNANA-based cleavage assay, and egress inhibition assay support that the somatically mutated mAbs FluA-168 and FluA-173 potently inhibit NA enzymatic activity by binding directly to the NA active site and preventing egress of nascent virions from infected cells.

### Small-molecule competition and structural analysis of FluA mAbs.

**Figure 3:**
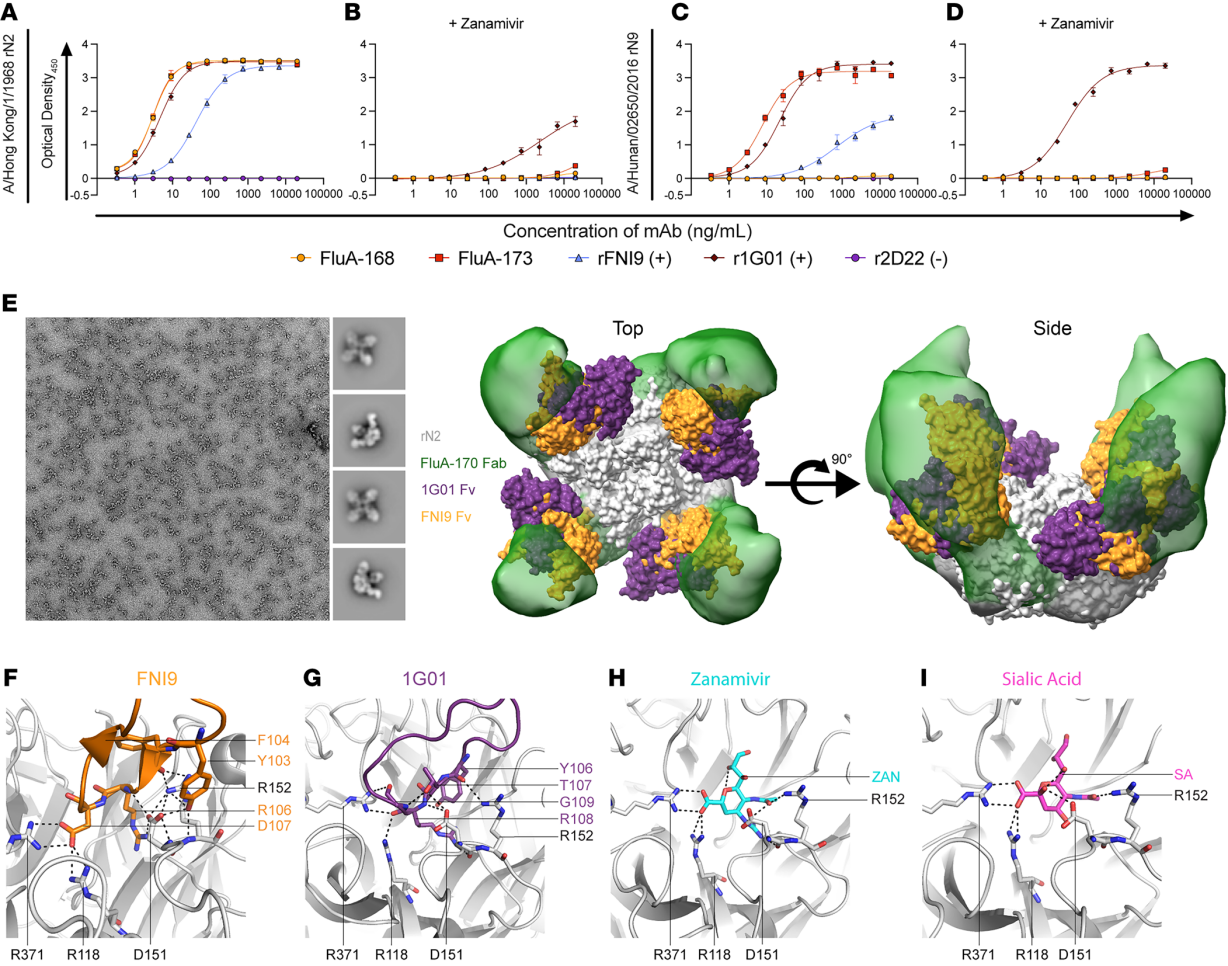
mAbs interact with conserved residues of the NA active site. (**A**–**D**) Zanamivir-competition ELISA showing binding reactivity of FluA-168 and FluA-173, positive control mAbs r1G01 and rFNI9, and isotype-negative control mAb r2D22 to rN2 and rN9 antigens in the presence of zanamivir. Data points and error bars indicate mean ± SD. All mAbs were assessed in triplicate across 2 biological replicates. (**E**) Negative stain electron microscopy (NS-EM) micrograph with 2D class averages shown. Original magnification ×50,000 with a pixel size of 2.18 Å per pixel. Top and side views of the NS-EM reconstruction of representative mAb FluA-170 Fab molecules in complex with one rN2 (gray; A/Singapore/1/1957) are shown. The Fv region of positive control mAbs 1G01 (purple; PDB ID: 6Q23) and FNI9 (orange; PDB ID: 8G30) is also shown in complex with the rN2 antigen. (**F**) Network of polar interactions between Y103, F104, R106, and D107 in the HCDR3 of FNI9 (orange) to R118, D151, R152, and R371 of N2 (gray) based on PDB: 8G30. (**G**) Network of polar interactions between Y106, T107, R108, and G109 in the HCDR3 of 1G01 (purple) to R118, D151, R152, and R371 of N1 (gray) based on PDB: 6Q23. (**H**) Network of polar interactions between zanamivir (ZAN; cyan) and R118, D151, R152, and R371 of N9 (gray) based on PDB: 4MWX. (**I**) Network of polar interactions between sialic acid (SA; pink) and R118, D151, R152, and R371 of N2 (gray) based on PDB: 4GZQ. Dashed lines indicate interactions within 3.5 Å.

To further deduce the contact residues of the NA active site recognized by mAbs of this B cell lineage, a zanamivir-competition ELISA was performed using representative rN2 and rN9 antigens A/Hong Kong/1/1968 and A/Hunan/02650/2016, respectively. In addition to r1G01, a recombinant version of the broadly neutralizing NA antibody FNI9 (rFNI9) was used as a control to assess sensitivity to zanamivir competition (32). The binding of FluA-168, FluA-173, and the active site mAb rFNI9 to rN2 was substantially diminished when the rN2 antigen was first saturated with zanamivir (Figure 3B) compared with the nonsaturated control (Figure 3A). Similarly, saturation of rN9 with zanamivir abrogated binding of FluA-168, FluA-173, and the active site mAb rFNI9 (Figure 3D) compared with the nonsaturated control (Figure 3C). The active site mAb r1G01, however, retained binding reactivity to both rN2 and rN9 but did exhibit a decrease in binding to the rN2 antigen (Figure 3, B and D). Together, these results further support that mAbs of this B cell lineage bind directly to the NA active site and likely interact with conserved residues shared by zanamivir and rFNI9.

We next used negative stain electron microscopy (NS-EM) to further define the site targeted by this lineage. Antigen-binding fragments (Fabs) of FluA-170 were recombinantly expressed and allowed to complex with the A/Singapore/1/1957 rN2 antigen. FluA-170 bound to the corners of the N2 tetramer, approaching from the top at an oblique angle (Figure 3E). This finding is consistent with previously reported NA-specific mAbs that bind near the active site and neutralize NA enzymatic activity (32). Overlaying the electron density localization of FluA-170 Fabs with surface representations of the variable regions (Fv) of mAbs 1G01 and FNI9 bound to rN2 demonstrated an overlap of known NA active mAbs with the representative FluA-170 of this B cell lineage. Moreover, we compared the resolved structures of 1G01, FNI9, zanamivir, and sialic acid in complex with rN1, rN2, or rN9 antigens to identify shared contact residues in the NA active site. Among these 3 NA subtypes, residues R118, D151, R152, and R371 made polar contacts with the known NA active site mAbs; the small-molecule inhibitor zanamivir; and the receptor of influenza virus, sialic acid (Figure 3, F–I). Jointly, these findings support the hypothesis that antibodies of this B cell lineage may interact with highly conserved NA active site residues. As in the case of 1G01 and FNI9, interactions with these residues likely explain the observed broad reactivity to the IAV N2 and N9 subtypes.

### H1N1 NA sialidase activity is inhibited by NA-reactive mAbs.

**Figure 4:**
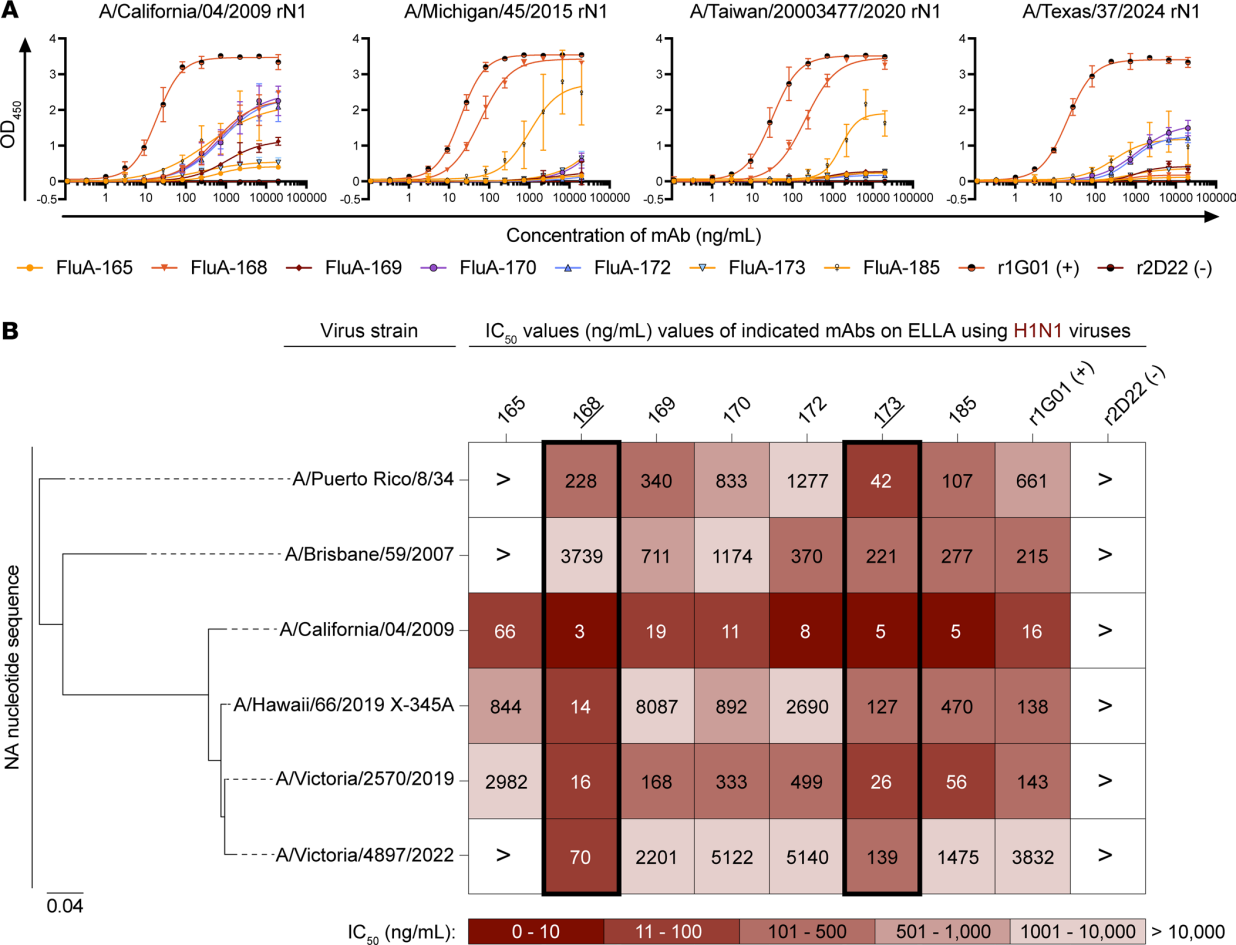
mAbs bind and inhibit the sialidase activity of the IAV N1 subtype. (**A**) ELISA curves indicating binding potency of downselected mAbs to rN1 antigens of different H1N1 strains. Data points and error bars indicate mean ± SD. All mAbs were assessed in triplicate across 2 biological replicates. (**B**) ELLA heatmap indicating the potency of mAb-mediated inhibition of NA sialidase activity measured on a panel of H1N1 viruses. Columns represent mAbs used, and rows represent indicated H1N1 virus strains. The potency of NA sialidase activity inhibition is represented as an IC_50_ value. Candidate mAbs FluA-168 and FluA-173 are highlighted. The phylogenetic tree indicates the relatedness of the NA nucleotide sequence between each IAV strain shown. Scale bar: average number of nucleotide substitutions between the indicated virus strains. All mAbs were assessed in triplicate across 2 biological replicates.

Prompted by the zanamivir competition data and shared active site residues between the N1, N2, and N9 IAV subtypes, we tested whether antibodies of the FluA B cell lineage could bind and inhibit the NA sialidase activity of the N1 subtype. Antibodies were tested for binding to stabilized rN1 antigens representative of seasonal H1N1 strains circulating during the years 2009–2015 and a highly pathogenic H5N1 strain (A/Texas/37/2024). Each mAb, particularly those previously downselected for functional assays, exhibited reactivity to one or more N1 strains (Figure 4A and Supplemental Figures 7 and 8). Moreover, the 7 previously downselected antibodies also showed inhibition of H1N1 viruses representing circulating strains from 1934 to 2022 (Figure 4B, Supplemental Figures 10 and 11). Candidate mAbs FluA-168 and FluA-173 exhibited potent inhibition of sialidase activity against the most modern strain, A/Victoria/4897/2022 (Figure 4B). These data — combined with functional data against the H3N2 and H7N9 virus strains — indicate that mAbs from this clonal lineage can potently inhibit the enzymatic function of NA across H1N1, H3N2, and H7N9 subtypes.

### FluA-168 and FluA-173 protect mice from a lethal H3N2 challenge through neutralization activity.

**Figure 5:**
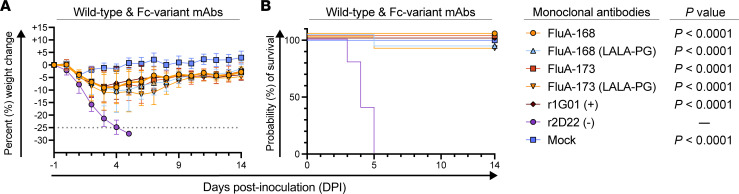
FluA-168 and FluA-173 protect against H3N2 virus lethality. (**A**) Weight loss and (**B**) Kaplan-Meier survival curves for mice immunized with WT or LALA-PG Fc variants from the day of immunization (day –1) to a humane end point (25% weight loss) or 14 days after inoculation. Mice immunized with the LALA-PG Fc variant mAbs of FluA-168 and FluA-173 had a 90% survival rate compared with WT (100%), and both had a significantly increased probability of survival compared with mice immunized with r2D22 (0%). *P* values were calculated using the log-rank (Mantel-Cox) test, and a Bonferroni-corrected statistical threshold was applied (α ≤ 0.008).

We next sought to assess the protective efficacy of FluA-168 and FluA-173 in a murine model of H3N2 infection. In this study, mice were passively immunized with one 10 mg/kg dose via i.p. injection 24 hours before intranasal (i.n.) inoculation with a lethal dose of a murine-adapted H3N2 virus (A/Aichi/2/1968 X-31). Mice were monitored for weight loss and survival. Positive and negative control groups were included and administered r1G01 and r2D22, respectively. A mock-treated control group was included that was sham-immunized and inoculated with a viral growth medium containing no virus. Both FluA-168 and FluA-173 completely protected against mortality (100%) and reduced weight loss (Figure 5, A and B) compared with the isotype-matched negative control (r2D22) treatment group (0%). These data indicated that these mAbs protect against IAV infection in vivo.

Influenza-specific mAbs can elicit Fc-mediated effector functions, such as antibody-dependent cellular cytotoxicity (ADCC) to protect against influenza infection (56). To assess whether the mechanism of protection of FluA-168 and FluA-173 is mediated solely by inhibition of NA enzymatic activity or if Fc effector functions are also involved, we generated versions of FluA-168 and FluA-173 in which previously described LALA-PG (L234A-L235A-P329G) substitutions were introduced into the Fc region (57, 58). These Fc variants of FluA-168 and FluA-173 retain their ability to bind to NA antigens but do not interact with Fcγ receptors or C1q (58). Mice immunized with WT FluA-168 and FluA-173 were completely protected (100%) from mortality, while 90% of the mice immunized with the LALA-PG Fc variant of either FluA-168 or FluA-173 survived (Figure 5, A and B). These observations suggest that at the tested dose, these mAbs primarily protect through inhibition of NA enzymatic activity; however, Fc-mediated effector functions may partially contribute to the overall protection observed against IAV infection (32).

## Discussion

NA-specific antibodies can impact the replication of influenza viruses in a number of different ways. mAbs that inhibit sialidase activity can prevent the virus from cleaving sialylated mucins, preventing the virus from migrating through the respiratory mucosa and infecting the respiratory epithelium (13, 14). Inhibition of NA sialidase activity can also abrogate viral egress from the surface of infected cells, preventing spread to other susceptible host cells (3, 5, 12, 30, 59). Finally, NA-specific mAbs can facilitate Fc-mediated effector functions to recruit immune cells that destroy infected host cells via ADCC and remove opsonized virus particles in the respiratory lumina (59, 60). All of these mechanisms likely play some role in the protective effects of NA antibodies observed in cohort studies and animal models, but the development and evolution of cross-reactivity in human antibody responses to IAV are not well understood.

In this study, we examined the human antibody response to IAV by isolating and characterizing NA-specific mAbs from an individual with an extensive influenza vaccination and natural infection history. We identified a clonally expanded B cell lineage that cross-reacted with and inhibited diverse viruses from N1, N2, and N9 IAV subtypes. Using phylogenetically inferred ancestral sequences, we showed that this lineage was likely elicited by infection during early childhood and acquired breadth through somatic mutations. We demonstrated through the use of competition-binding and structural techniques that this cross-reactivity was likely due, in part, to members of this lineage making contacts with highly conserved residues in the NA active site. Finally, we demonstrated that 2 mAbs from this lineage protected mice from infection and that this protection was largely independent of engagement of Fc effector functions.

Antibodies of the clonally expanded B cell lineage are encoded by the heavy- and light-chain variable genes IGHV1-18*04 and IGKV1-5*01, respectively. To our knowledge, an NA-specific mAb encoded by *IGHV1-18* that targets the NA active site has not been reported, suggesting that these mAbs belong to an individual-specific antibody lineage. Several mAbs have been described that broadly target the active site of either IAV or IBV NA glycoproteins, including some that react to both IAV and IBV NA (30–32, 53, 61–64). However, whether B cells with cross-reactive specificities exist at low levels or develop over repeated exposures remains largely understudied.

All mAbs and their inferred UCAs reacted to rN2 antigens that the donor may have been exposed to over their lifetime; however, many mAbs lost reactivity to circulating N2 strains as time progressed, consistent with antigenic drift abrogating antibody binding. Importantly, these antibodies were derived from plasmablasts elicited in response to an acute infection in 2017, despite many of these antibodies losing binding reactivity against NA strains after 2014. This is an example of immune imprinting, a phenomenon also known as “original antigenic sin,” in which antibody specificities established by prior exposures may be recalled even if they have low affinity for the current antigen (6, 28, 37, 65–69). While cross-reactive and protective mAbs, such as FluA-168 and FluA-173, may be boosted during vaccination or infection, other less cross-reactive mAbs would likely not be as useful. We also note that the evolution of cross-reactivity against N1, N2, and N9 in this lineage appears to be a complex process. mAbs with cross-reactivity to the IAV N9 subtype were first identified after a natural H3N2 infection in 2017 and again following a second H7N9 vaccination in 2019 (Supplemental Figure 5, A and B) (70). While we cannot definitively link the emergence of reactivity to the IAV N9 subtype to a prior H7N9 vaccination in 2013, the expansion of this B cell lineage in response to these heterologous exposures emphasizes the importance of understanding how broadly reactive B cells are elicited and recalled as individuals are repeatedly exposed to IAV antigens through infection or vaccination (3, 71–73). Based on binding reactivity and sialidase inhibition assays, several of these broadly cross-reactive antibodies exhibited potent inhibition of H1N1, H3N2, and H7N9 virus subtypes. However, some mAbs that cross-reacted with the N9 subtype were poorly reactive to contemporary N2 strains, while other mAbs maintained cross-reactivity with recent N2 strains but also acquired N9 reactivity. This may suggest that cross-reactivity requires not only somatic mutations that introduce additional contacts but also ones that avoid steric clashes. Given the extraordinary breadth observed for some NA active site antibodies, future studies that dissect how cross-reactivity evolves could prove useful for reverse-vaccinology approaches to elicit NA active site–directed immunity through vaccination.

This study contains several limitations. First, the investigation focused on one healthy male donor following vaccination or natural infection, which cannot fully recapitulate the intricacy of the human antibody response to all vaccination or infection scenarios. The study also observed one clonally expanded B cell lineage over several years and did not account for the proportion of the individual’s immune response comprising this clonal family, the presence of other such B cell lineages, or the frequency at which similar clonotypes occur in the human population. The study also used 4 rN1 antigens and 1 rN9 antigen to test for cross-reactivity and does not address the ability of these mAbs to protect from an H1N1 or H7N9 infection in vivo. Future studies could include additional NA antigens and relevant H1N1 and H7N9 virus strains for in vivo protection studies to address the ability of cross-reactive clonotypes to broadly protect against 3 distinct IAV subtypes.

Together, the results of this study shed light on the evolution of cross-reactivity in a B cell lineage targeting the active site of IAV NAs and underscore the importance of NA-mediated humoral immunity in reducing the disease burden associated with IAV infection. The annual influenza vaccine remains the most accessible mitigation strategy against influenza, albeit with varied effectiveness between seasons (3, 74, 75). While the discovery and engineering of broadly cross-reactive and neutralizing antibodies against influenza could lead to the development of antibody-based therapeutics, vaccine-based approaches will require the design of stabilized NA immunogens that, when administered prophylactically, elicit robust NAI antibody responses to circulating IAV and IBV strains. Studies like ours that examine the evolution of cross-reactivity in B cell responses to distinct influenza antigens will further these efforts to develop an influenza vaccine that broadly protects against symptomatic disease (28, 47, 48, 76).

## Methods

### Sex as a biological variable.

mAbs isolated and expressed in this study were derived exclusively from one healthy male donor. Sex was not considered as a biological variable during donor recruitment, and it is unknown whether these findings reflect the heterogeneity of the influenza-specific humoral response found in female individuals. Furthermore, this study exclusively examined protection from lethality in female mice. Prior studies suggest that female mice have a more robust humoral response to influenza vaccination and infection compared with male mice and may exhibit greater protection from morbidity after challenge with a sublethal dose of influenza (77–79). Thus, passive transfer of mAbs that protect from a lethal dose of influenza in a female mouse is likely to protect against lethality in male mice.

### Cell lines.

Standard Madin-Darby canine kidney (MDCK) cells were obtained by ATCC (catalog CCL-34) and cultured in Dulbecco’s Minimal Essential Medium (DMEM), high glucose, GlutaMAX Supplement (Gibco, catalog 10566016) with 10% heat-inactivated fetal bovine serum (HI-FBS; Gibco, catalog 16250078) and 1% penicillin-streptomycin-glutamine (Pen-Strep-Glutamine, Gibco, catalog 10378016) and incubated at 37°C in 5% CO_2_. The variant MDCK cell line, MDCK-SIAT1, derived by stable transfection of MDCK cells with the cDNA of human 2,6-sialtransferase (SIAT1), was obtained from Sigma-Aldrich (catalog 05071502-1VL). MDCK-SIAT1 cells were cultured in DMEM (Gibco, catalog 11965092) supplemented with 10% HI-FBS (Gibco, catalog 38401-02), 1.25% of 1 M HEPES buffer (Gibco, catalog 15630080), 1.25% sodium pyruvate (Gibco, catalog 11360070), and 1% Pen-Strep (Gibco, catalog 15140122) and incubated at 37°C in 5% CO_2_. ExpiCHO (hamster, female origin) and FreeStyle 293F cell lines were purchased (Thermo Fisher Scientific) and cultured according to the manufacturer’s protocol. All cell lines were tested for mycoplasma monthly, and all cell lines tested negative during the time of the study.

### H1N1 viruses.

Seed stocks of A/Puerto Rico/8/1934, A/Brisbane/59/2007, A/California/04/2009, A/Hawaii/66/2019 X-345A, A/Victoria/2570/2019, and A/Victoria/4897/2022 were obtained from the International Reagent Resource (IRR) or the National Institute for Biological Standards and Control (NIBSC). Working stocks of A/Puerto Rico/8/1934, A/Brisbane/59/2007, A/California/04/2009, A/Hawaii/66/2019 X-345A, A/Victoria/2570/2019, and A/Victoria/4897/2022 were made by propagating and titrating each virus in monolayer cultures of MDCK or MDCK-SIAT1 cells using DMEM supplemented with 1.25% of 1 M HEPES buffer, 1.25% of 7.5% BSA, 1% Pen-Step, and 1 μg/mL TPCK-treated trypsin [Sigma-Aldrich, catalog T1426-10MG]). Cells were maintained in DMEM supplemented with 10% HI-FBS, 1.25% of 1 M HEPES buffer, 1.25% sodium pyruvate, and 1% Pen-Strep. Prior to inoculation, cell monolayers were washed with Dulbecco’s Phosphate-Buffered Saline (DPBS, 1× without Ca^2+^ & Mg^2+^; Corning, catalog 21-031-CM) to remove residual FBS. A working stock of A/Victoria/2570/2019 was made by propagating in embryonated chicken eggs (Charles River Laboratories). All viruses were manipulated under BSL-2 conditions.

### H3N2 viruses.

Seed stocks of A/Hong Kong/1/1968, A/Aichi/2/1968 X-31, A/Victoria/3/1975, A/Beijing/353/1989, A/Sydney/5/1997, A/Tasmania/503/2020, and A/Darwin/6/2021 were obtained from the International Reagent Resource (IRR). Working stocks of each virus were made by propagating and titrating each virus in monolayer cultures of MDCK-SIAT1 cells using DMEM supplemented with 1.25% of 1 M HEPES buffer, 1.25% of 7.5% bovine serum albumin [BSA], 1% Pen-Step, and 1 μg/mL TPCK-treated trypsin [Sigma-Aldrich, catalog T1426-10MG]). Cells were maintained in DMEM supplemented with 10% HI-FBS, 1.25% of 1 M HEPES buffer, 1.25% sodium pyruvate, and 1% Pen-Strep. Prior to inoculation, cell monolayers were washed with Dulbecco’s Phosphate-Buffered Saline (DPBS, 1× without Ca^2+^ & Mg^2+^, Corning, catalog 21-031-CM) to remove residual FBS. All viruses were manipulated under BSL-2 conditions.

### H7N9 virus.

A reverse genetics-derived H7N9 virus expressing the H7 and N9 genes of A/Shanghai/2/2013 on a PR8 (A/Puerto Rico/8/1934 [H1N1]) backbone, designated A/Shanghai/2/2013-PR8-IDCDC-RG32A, was obtained from IRR (catalog FR-1389). Working stocks of the virus were made by propagating the virus in embryonated chicken eggs (Charles River Laboratories). The virus was titrated in monolayer cultures of MDCK cells using DMEM supplemented with 2% BSA and 1 μg/mL TPCK-treated trypsin. Cells were maintained in DMEM, high glucose, GlutaMAX Supplement with 10% HI-FBS and 1% Pen-Strep-Glutamine. Prior to inoculation, cell monolayers were washed with DMEM supplemented with 2% BSA to remove residual FBS. A/Shanghai/2/2013-PR8-IDCDC-RG32A was manipulated under BSL-2 conditions with BSL-3 practices.

### Isolation of PBMCs.

Procedures for isolation of PBMCs and flow cytometric sorting of plasmablasts have been previously described (49). Peripheral blood samples were collected 7 and 28 days after immunization with the seasonal influenza vaccine from an otherwise healthy individual with an extensive history of annual seasonal influenza vaccinations, two prior experimental H5N1 vaccinations, and two H7N9 vaccinations in the context of vaccine clinical trials. Additional peripheral blood was collected 7 days after the onset of symptoms associated with a natural, laboratory-confirmed IAV H3N2 infection. PBMCs were isolated by Ficoll density gradient centrifugation, cryopreserved, and stored in liquid nitrogen until use unless otherwise specified.

### Single-cell sequencing of antibody variable genes following a natural influenza infection.

The techniques used for RNA isolation and sequencing of antibody variable genes from individual plasmablasts was previously described (49, 80–83). Briefly, PBMCs from the infected individual were stained in cell sorting buffer (DPBS supplemented with 2% HI-FBS and 1 mM EDTA) with the following phenotyping antibodies: anti-CD19–FITC (1:20 dilution, eBioscience, 11-0199-42), anti-CD27–APC (1:20 dilution, BD Biosciences, 558664), and anti-CD38–PE (1:25 dilution, BD Biosciences, 555460). Cells were also stained with propidium iodide to assess viability. Stained cells were sorted into sc-V_H_/V_L_Seq sequencing buffer for single-cell RNA sequencing using the 10x Genomics Chromium platform with enrichment by the 5′ VDJ amplification kit (10x Genomics) following the manufacturer’s recommendations.

### Single-cell sequencing of antibody variable genes following influenza vaccination.

PBMCs from the vaccinated individual were enriched for plasmablasts using a custom plasmablast enrichment kit (StemCell, catalog 19309). Cells were then stained in cell sorting buffer (DPBS supplemented with 2% HI-FBS and 1 mM EDTA) with the following phenotyping antibodies: anti-CD19–FITC (1:20 dilution, eBioscience, 11-0199-42), anti-CD27–APC (1:20 dilution, BD Biosciences, 558664), and anti-CD38–PE (1:25 dilution, BD Biosciences, 555460). Cells were also stained with DAPI to assess viability. Stained cells were sorted into sc-V_H_/V_L_Seq sequencing buffer for single-cell RNA sequencing using the 10x Genomics Chromium platform with enrichment by the 5′ VDJ amplification kit (10x Genomics) following the manufacturer’s recommendations.

### Antibody variable gene sequence analysis.

B cells of interest were processed through the 10x Genomics workflow, and sequences were further processed using Cell Ranger software v3.1.0 (10x Genomics). All sequences were analyzed using PyIR software (84) to identify the variable (V) gene, joining (J) gene, and the third complementarity region (CDR3) sequence of each antibody variable region, as previously described (85). Annotated sequence data were stored in a MongoDB database for further study. Antibodies of interest were identified based on (i) matching *IGHV* and *IGLV* gene usage and (ii) 80% nucleotide sequence identity of the heavy-chain CDR3 (HCDR3). All antibodies whose heavy- and light-chain sequences matched these criteria were defined as members of a B cell lineage and used for recombinant expression.

### Phylogenetic analysis of recombinantly expressed antibodies and NA antigens.

For the antibody phylogenetic trees shown in Figure 1C, Figure 2A, and Figure 4B, antibody heavy-chain variable gene sequences were first aligned to their corresponding germline gene using Muscle v5 (86, 87). Neighbor-joining trees of the heavy chains from the aligned sequences were then made using the Geneious Tree Builder in Geneious Prime v2025.1.2 with the respective germline *IGHV* gene as an outgroup (88). The resultant phylogenetic trees were visualized using Geneious Prime v2025.1.2. The inferred UCAs were identified using the Augur toolkit (89). Similarly, the native sequences of the IAV N1 and N2 subtypes were aligned to their corresponding ancestral NA sequence using Muscle v5 (86, 87). Neighbor-joining trees of the aligned NA sequences were made using the Geneious Tree Builder in Geneious Prime v2025.1.2 and were rooted to their corresponding ancestral NA sequence. The resultant phylogenetic trees of the IAV N2 and N1 subtypes were visualized using Geneious Prime v2025.1.2 as shown in Figure 2A and Figure 4B, respectively.

### Phylogenetic analysis of a B cell lineage.

For the antibody phylogenetic trees shown in Supplemental Figure 4, Supplemental Figure 5B, and Supplemental Figure 6, antibody heavy-chain variable gene sequences were first aligned to FluA-173 using the MAFFT method in the Augur toolkit (89, 90) to avoid trimming full-length gene sequences. A maximum-likelihood tree was then generated using the IQ-TREE method in the Augur toolkit (89, 91) and rooted to the respective germline *IGHV* gene. The resultant tree was visualized using the Auspice visualization tool via Nextstrain (92) and colored based on the first year in which the sequence was acquired. The phylogenetic tree in Supplemental Figure 5B is filtered to focus on the panel of 22 recombinantly expressed mAbs from the FluA B cell lineage. Similarly, the phylogenetic tree in Supplemental Figure 6 is filtered to focus on those recombinantly expressed mAbs that are reactive to the IAV N9 subtype based on ELISA binding reactivity, as shown in Figure 1C.

### Recombinant mAb production and purification.

Paired heavy- and light-chain antibody variable gene sequences were synthesized as cDNA and cloned into a human IgG1 expression vector by Twist Biosciences. This vector contains 2A peptide sequences and a Gly-Ser-Gly amino acid linker that allows for the expression of antibody heavy and light-chain genes from the same construct when transfected in mammalian cell culture (93). We previously described the microscale expression of mAbs in 1 mL ExpiCHO cell cultures (Thermo Fisher Scientific, catalog A29127) in 96-well plates (50, 80). For larger-scale mAb expression, we transfected (1–300 mL cultures of ExpiCHO cells per antibody) following the Gibco ExpiCHO Expression System (Thermo Fisher Scientific) protocol for 50 mL mini-bioreactor tubes (Corning). Culture supernatants were purified using HiTrap MAbSelect SuRe resin (Cytivia, formerly GE Healthcare Life Sciences) on a 24-column parallel protein chromatography system (Protein BioSolutions). Purified mAbs were buffer-exchanged into PBS, concentrated using Amicon Ultra-4 50-kDa centrifugal filter units (Millipore Sigma), and stored at 4°C until use.

### Recombinant antigen production and purification.

Soluble, recombinant NA (rNA) antigens were expressed and purified as previously described (80). Genes encoding the NA of IAV strains A/Singapore/1/1957 (H2N2 NA, GenBank: CY125896.1), A/Hong Kong/1/1968 (H3N2 NA, GenBank: CY112251.1), A/Bangkok/1/1979 (H3N2 NA, GenBank: CY114431.1), A/Memphis/7/1985 (H3N2 NA, GenBank: CY008710.1), A/Johannesburg/33/1995 (H3N2 NA, GenBank: CY121343.1), A/Minnesota/11/2010 (H3N2 NA, GenBank: KJ942626.1), A/Fujian/411/2002 (H3N2 NA, GenBank: CY112935.1), A/Brisbane/10/2007 (H3N2 NA, GenBank: EU199249.1), A/Hong Kong/4801/2014 (H3N2 NA [N245/T247], GISAID accession: EPI1868574), A/Colorado/15/2014 (H3N2 NA [S245/S247], GISAID accession: EPI926148), A/Tennessee/3/2019 (H3N2 NA, GISAID accession: EPI1363096), A/Darwin/11/2021 (H3N2 NA, GISAID accession: EPI1859985), A/California/04/2009 (H1N1 NA, GenBank: MN371610.1), A/Michigan/45/2025 (H1N1 NA, GenBank: MK622934.1), A/Taiwan/20003477/2020 (H1N1 NA, GISAID accession: EPI3907986), A/Texas/37/2024 (H5N1 NA, GISAID accession: EPI3171486), and A/Hunan/02650/2016 (H7N9 NA, GISAID accession: EPI961189) were codon-optimized for mammalian cell expression, and cDNAs were synthesized and cloned into a DNA plasmid expression vector. Each recombinant antigen contained an IL-2 signal peptide sequence, an 8x His-tag, an AviTag site-specific biotinylation sequence, a human vasodilator-stimulated phosphoprotein (hVASP) tetramerization domain, a thrombin cleavage site, a short linker sequence, and the ectodomain from the NA of the indicated IAV strain. Additional mutations were included in each rN1 antigen to stabilize its closed tetrameric conformation as previously described (94). Antigens were expressed by transient transfection of FreeStyle 293-F cells (Gibco, catalog R79007) or Expi293F cells (Gibco, catalog A14527). Cell supernatants were collected after seven days, sterilized by filtration with a 0.4 μm filter, and purified using a HisTrap Excel (Cytiva, catalog 17-3712-05) column.

### ELISA.

384-well plates were coated with 2 μg/mL purified rNA protein in 1× DPBS and incubated at 4°C overnight. Plates were washed 3 times with DPBS containing 0.05% Tween-20 (DPBS-T) and incubated with blocking buffer (DPBS with 2% nonfat dry milk, 2% heat-inactivated goat serum, and 0.05% Tween-20) at room temperature (RT) for 1 hour. Primary mAbs were diluted in blocking buffer at a starting concentration of 20 μg/mL and serially diluted 3-fold. A recombinantly-expressed human anti-dengue virus mAb (r2D22) was used as an isotype-matched negative control (51). Plates were washed 3 times with DPBS-T before adding primary mAbs to the appropriate wells and incubating at RT for 1 hour. A secondary goat anti-human IgG antibody conjugated with HRP (Southern Biotech, catalog 2014-05) was diluted 1:5,000 in blocking buffer immediately before use. Plates were washed 3 times with DPBS-T before adding the secondary antibody to all wells and incubating at RT for 1 hour in the dark. Plates were washed 3 times before adding 25 μL of a 3,3′,5,5′-tetramethylbenzidine (TMB) substrate (Thermo Fisher Scientific, catalog PI34029) to all wells. Color development was monitored in the dark, and 25 μL of 1 M hydrochloric acid (HCl) was added to stop the reaction before measuring optical density at 450 nm (OD_450_) using a spectrophotometer (BioTek). EC_50_ values were determined in Prism v10 (GraphPad) using sigmoidal dose-response nonlinear regression analysis. ELISAs were performed in technical triplicate and biological duplicate.

### Zanamivir-competition ELISA.

384-well plates were coated with 2 μg/mL purified rNA protein in 1× DPBS and incubated at 4°C overnight. Plates were washed 3 times with DPBS-T and incubated with blocking buffer at RT for 1 hour. Zanamivir was diluted in blocking buffer at a concentration of 40 μg/mL. Plates were washed 3 times with DPBS-T before adding diluted zanamivir to the appropriate wells and incubating at RT for 30 minutes. Blocking buffer containing no zanamivir was added to additional wells of the same plate as a control. Primary mAbs were diluted in blocking buffer at a starting concentration of 40 μg/mL and serially diluted three-fold. Antibody r2D22 was included as an isotype-matched negative control (51). Primary mAbs were added to the appropriate wells containing zanamivir or control wells containing no zanamivir (final mAb concentration, 20 μg/mL) and incubated at RT for 1 hour. A secondary goat anti-human IgG antibody conjugated with HRP (Southern Biotech, catalog 2014-05) was diluted 1:5,000 in blocking buffer immediately before use. Plates were washed 3 times with DPBS-T before adding the secondary antibody to all wells and incubating at RT for 1 hour in the dark. Plates were washed 3 times with DPBS-T before adding 25 μL of a TMB substrate (Thermo Fisher Scientific, catalog PI34029) to all wells. Color development was monitored in the dark, and 25 μL of 1 M HCl was added to stop the reaction before measuring OD_450_ using a spectrophotometer (BioTek). EC_50_ values were determined in Prism v10 (GraphPad) using sigmoidal dose-response nonlinear regression analysis. ELISAs were performed in technical triplicate and biological duplicate.

### ELLA.

384-well plates were coated with 25 μg/mL fetuin (Sigma-Aldrich, catalog F2379-100MG) in 25 μL of 1× DPBS at 4°C for at least 18 hours. Before performing this assay, each virus was titrated to determine the highest dilution factor that yields its maximum OD_450_. Primary mAbs were diluted in sample diluent (1X DPBS with CaCl_2_ and MgCl_2_ [Corning, catalog 21-030-CM], 1% BSA, and 0.5% Tween-20) at a starting concentration of 20 μg/mL and serially diluted 3-fold. Antibody r2D22 was used as an isotype-matched negative control (51). Plates were washed 3 times with DPBS-T before adding 25 μL primary mAbs to the appropriate wells, excluding virus-only and virus-free control columns. Next, 25 μL diluted virus was added to all wells except for the virus-free column (final mAb concentration, 10 μg/mL). Additional sample diluent (25 μL) was added to each well of the virus-free column. Plates were placed in a humidified incubator at 37°C for 18 hours. A PNA-HRP (Sigma-Aldrich, catalog L7759-1MG) enzyme was diluted to 1 μg/mL in DPBS with 1% BSA immediately before use. Plates were washed six times with DPBS-T before adding 25 μL diluted PNA-HRP to each well and incubating at RT for 2 hours in the dark. Plates were washed 3 times with DPBS-T before adding 25 μL of a TMB substrate (Thermo Fisher Scientific, catalog PI34029) to all wells. Color development was monitored in the dark. The reaction was stopped with 25 μL of 1 M HCl, and the OD_450_ of each well was measured using a spectrophotometer (BioTek). The data were normalized in Excel (Microsoft) to the virus-only control to identify the percentage of sialidase activity inhibited at each mAb concentration. Half-maximal inhibitory concentration (IC_50_) values were determined in Prism v10 (GraphPad) using sigmoidal dose-response nonlinear regression analysis. The IC_50_ value was defined as the concentration of mAb at which 50% of sialidase activity was inhibited compared with the negative (virus-free) control. ELLAs were performed in technical triplicate and biological duplicate.

### MUNANA-based assay.

The NA-Fluor Influenza Neuraminidase Assay Kit (Thermo Fisher Scientific, catalog 4457091) was used to quantify NA activity as measured by cleavage of the MUNANA substrate. Before performing this assay, each virus was titrated to determine the highest dilution factor that yields its maximum relative fluorescence units (RFU). The experiments were performed according to the manufacturer’s protocol. Primary mAbs were diluted in Assay Buffer at a starting concentration of 20 μg/mL and serially diluted three-fold. Antibody r2D22 was used as an isotype-matched negative control (51). Next, 6.25 μL diluted primary mAb was added to the appropriate wells of a black, flat-bottom 384-well plate, excluding virus-only and virus-free control columns. An equal volume (6.25 μL) of diluted virus was added to each well of the black, flat-bottom 384-well plate except for one virus-free column (final mAb concentration,10 μg/mL). As a virus-free control, 6.25 μL NA-Fluor Assay Buffer was added to each well of the virus-free column. Plates were placed in a humidified incubator at 37°C for 30 minutes. After incubation, 12.5 μL NA-Fluor Substrate was added to all wells and placed in a humidified incubator at 37°C for 1 hour. The reaction was stopped with 25 μL NA-Fluor Stop Solution, and the fluorescence intensity of each well was measured using a spectrophotometer (BioTek) after exciting at 360 nm and detecting emission at 450 nm. The data were normalized in Excel (Microsoft) to the virus-only control to identify the percentage of sialidase activity inhibited at each mAb concentration. All IC_50_ values were determined in Prism v10 (GraphPad) using sigmoidal dose-response nonlinear regression analysis. The IC_50_ value was defined as the concentration of mAb at which 50% of MUNANA cleavage was inhibited. The MUNANA-based assay was performed in technical triplicate and biological triplicate.

### H3N2 egress inhibition assay.

MDCK-SIAT1 cells were diluted in cell growth medium at a concentration of 4.4 × 10^5^ cells/mL. Next, 100 μL of the cell suspension was added to each well of a 96-well TC-treated microplate (Corning, catalog CLS3997) for a seeding density of approximately 44,000 cells/well and incubated at 37°C in 5% CO_2_ overnight. Cells were then washed 3 times with a viral growth medium (DMEM supplemented with 1.25% of 1 M HEPES buffer, 1.25% of 7.5% BSA, 1% Pen-Step, and 1 μg/mL TPCK-treated trypsin [Sigma-Aldrich, catalog T1426-10MG]) to remove any residual FBS, inoculated with 100 μL virus at an MOI of 1 in the viral growth medium, and incubated at RT for 3 hours. A virus-free negative control column was included. Following inoculation, the cells were washed with viral growth medium and replenished with viral growth medium containing 3-fold serial dilutions of candidate mAbs, starting at 10 μg/mL. Antibody r2D22 was used as an isotype-matched negative control (51). Plates were incubated at 37°C in 5% CO_2_ overnight (~21 hours). Cell supernatants were collected and centrifuged for use in a hemagglutination assay. Turkey RBCs (LAMPIRE Biological Laboratories, catalog 7209403-50ML) were washed and diluted to 1% in DPBS. Equivalent volumes of MDCK-SIAT1 cell supernatant (50 μL) and 1% RBCs (50 μL) were combined in individual wells of a Nunc 96-well polystyrene conical bottom MicroWell plate (catalog 249952) and incubated at RT for 45 minutes. The maximal inhibitory concentration (IC_100_) value was defined as the lowest concentration of mAb at which no hemadsorption of RBCs occurred (i.e., no virus is present in the supernatant). The H3N2 egress inhibition assay was performed in technical duplicate and biological duplicate.

### H7N9 egress inhibition assay.

MDCK cells were diluted in cell growth medium at a concentration of 4 × 10^5^ cells/mL. Next, 100 μL of the cell suspension was added to each well of a 96-well TC-treated microplate (Corning, catalog CLS3997) for a seeding density of approximately 40,000 cells/well and incubated at 37°C in 5% CO_2_ overnight. Cells were then washed 3 times with DMEM supplemented with 2% BSA to remove any residual FBS, inoculated with 100 μL virus at an MOI of 1 in a viral growth medium (DMEM supplemented with 2% BSA and 1 μg/mL TPCK-treated trypsin), and incubated at RT for 3 hours. One column was inoculated with the viral growth medium containing no virus as a negative control. Following inoculation, the cells were washed 3 times with DMEM supplemented with 2% BSA and replenished with the viral growth medium containing three-fold serial dilutions of candidate mAbs, starting at 10 μg/mL. Antibody r2D22 was used as an isotype-matched negative control (51). Plates were incubated at 37°C in 5% CO_2_ overnight (~21 hours). Cell supernatants were collected and centrifuged for use in a hemagglutination assay. Turkey RBCs (LAMPIRE Biological Laboratories, catalog 7209403-50ML) were washed and diluted to 0.5% in DPBS. MDCK cell supernatant (25 μL) was diluted in 25 μL of 1× DPBS and then combined with 0.5% RBCs (50 μL) in individual wells of a Nunc 96-well polystyrene conical bottom MicroWell plate (catalog 249952) and incubated at RT for 45 minutes. The IC_100_ value was defined as the lowest concentration of mAb at which no hemadsorption of RBCs occurred (i.e., no virus is present in the supernatant). The H7N9 egress inhibition assay was performed in technical triplicate and biological duplicate.

### NS-EM.

Immune complexes were prepared by incubating rNA protein (A/Singapore/1/1957 H2N2, GenBank: CY125896.1) with recombinantly expressed FluA-170 Fab molecules at an antigen/Fab molar ratio of 1:4 at ambient temperature for 1 hour. Sample sizes of 3 μL at approximately 10–15 μg/mL were deposited on a carbon-coated 400 mesh copper grid (Electron Microscopy Sciences, catalog CF300-CU-50) that had been glow-discharged for 120 seconds. Grids were stained with 2% uranyl formate, and images were recorded on a Gatan US4000 4k × 4k CCD camera using an FEI TF20 (TFS) transmission electron microscope operated at 200 keV and controlled with SerialEM. All images were taken at 50,000× magnification with a pixel size of 2.18 Å per pixel in low-dose mode at a defocus of 1.5–1.8 μm. The total dose for the micrographs was between 25 and 30 e^–^/Å^2^. Image processing was performed using the CryoSPARC software package. Images were imported and CTF-estimated before selecting particles. Chosen particles were extracted using a box size of 160 pixels and binned by 2.5 (5.45 Å/pixel). Two-dimensional (2D) class averages were performed, and good classes were selected for ab initio model building and NU refinement using symmetry with a final resolution of approximately 27 Å. Docking of the model to the EM map was performed in ChimeraX v1.8. Surface representations of the variable fragments (Fvs) of the broadly neutralizing NA mAbs 1G01 (Protein Data Bank [PDB]: 6Q23) and FNI9 (PDB: 8G30) were overlayed with the electron density localization of FluA-170 Fab in ChimeraX v1.8 (95–97).

### Identifying conserved contact residues of N1, N2, and N9 IAV subtypes.

Resolved structures of NA active site antibodies 1G01 (PDB ID: 6Q23) and FNI9 (PDB: 8G30), the small-molecule inhibitor zanamivir (PDB: 4MWX), and the influenza receptor sialic acid (PDB: 4GZQ) in complex with rNA proteins were observed in PyMOL v3.1 (98). Conserved residues were identified by determining NA residues within 5.0 Å of any residues or atoms in FNI9, 1G01, zanamivir, or sialic acid. Only those NA residues that were shared between N1, N2, and N9 are shown.

### In vivo protective efficacy in a lethal H3N2 murine model.

Six- to 8-week-old female BALB/c mice were purchased from The Jackson Laboratory (strain 000651). Mice were housed in groups of ≤5 mice per cage at 18°C to 24°C ambient temperature and 40%–60% humidity. Cages were kept in individually ventilated cage racks with negative pressure ventilation and air filtering. A diet of 20% protein and 4.5% fat (LabDiet 5053, PicoLab Rodent Diet 20) was provided, and the mice were maintained on a 12-hour light/12-hour dark cycle (06:00 to 18:00). Food and water were available ad libitum. Mice were passively immunized with 10 mg of mAb per 1 kg of body weight via i.p. injection 24 hours before i.n. inoculation with virus. Antibody r2D22 was used as an isotype-matched negative control (51), and DPBS was administered as a mock treatment group. In ABSL-2 facilities, mice were anesthetized with a mixture of ketamine and xylazine via i.p. injection and inoculated i.n. with 6.5 × 10^5^ PFU/mouse of a murine-adapted IAV H3N2 strain (A/Aichi/2/1968 X-31). Mice were weighed and monitored daily for morbidity, and those losing ≥25% of initial body weight were humanely euthanized as per IACUC requirements. Data were analyzed in Prism v10 (GraphPad), and survival data were expressed as Kaplan-Meier curves. *P* values were calculated using the log-rank (Mantel-Cox) test, and a Bonferroni-corrected statistical significance threshold was applied (α ≤ 0.008) to account for multiple comparisons.

### Statistics.

Statistical analyses were performed using Prism v10 (GraphPad). Data presented in dose-response curves are shown as the mean ± SD. All EC_50_ and IC_50_ values were calculated from sigmoidal dose-response curves fit to data using nonlinear regression (Prism v10). Comparisons between the IC_100_ value of each mAb used in the viral egress assays (Figure 2C) were evaluated using the Kruskal-Wallis test with Dunn’s multiple comparison post hoc test. Differences in the probability of survival between each immunization group (Figure 5B) presented in Kaplan-Meier survival curves were evaluated using the log-rank (Mantel-Cox) test, and a Bonferroni-corrected statistical threshold was applied (α ≤ 0.008) to account for multiple comparisons.

### Study approval.

Studies involving human participants were approved by the Vanderbilt University Medical Center Institutional Review Board. Written informed consent was obtained prior to participation in this study. Details pertaining to sample collection are provided in *Isolation of PBMCs*. For animal experiments, BALB/c mice were purchased from The Jackson Laboratory (strain 000651). Breeding, maintenance, and experimentation complied with Vanderbilt Institutional Animal Care and Use Committee regulations and were performed under IACUC protocol M1900003-01. Details pertaining to mouse husbandry are provided in *In vivo protective efficacy in a lethal H3N2 murine model*.

### Data availability.

All data needed to evaluate the conclusions are presented in the supplemental material. Values for all data points in graphs and figures are reported in the Supporting Data Values file. The code used for sequence clustering is available on GitHub (https://github.com/crowelab/ibvna-paper-scripts/commit/729e41e). The sequences of FluA-164–FluA-185, including both UCAs, have been deposited to Genbank (PV941066–PV941113). Data and files necessary to replicate the phylogenetic trees shown in this article have been deposited to FigShare (DOI: https://doi.org/10.6084/m9.figshare.29473373.v1). Materials described in this article are available for distribution for nonprofit use using templated documents from the Association of University Technology Managers “Toolkit MTAs,” available at https://autm.net/surveys-and-tools/agreements/material-transfer-agreements/mta-toolkit Further information and requests for additional resources and reagents should be directed to the corresponding author.

## Author contributions

TAS, JEC, and SJZ conceptualized the study. TAS, RMW, IMG, LM, ECC, LEW, JEC, and SJZ developed the methodology. TAS, RMW, IMG, EB, RI, PG, RSN, RES, BNH, AT, KEW, CK, EV, LSH, and SJZ performed the investigations. TAS, JEC, and SJZ wrote the original draft of the manuscript. All authors edited and approved the final version. TAS and JEC acquired funding. JEC and SJZ supervised the study.

## Funding support

This work is the result of NIH funding, in whole or in part, and is subject to the NIH Public Access Policy. Through acceptance of this federal funding, the NIH has been given a right to make the work publicly available in PubMed Central.

- National Cancer Institute, NIH, Cancer Center Support grant P30CA068485, to Translational Pathology Shared Resource, VUMC.
- NIH, training grant 1 T32 AI112541-01A1, to TAS.
- NIH, 7K01 OD036063-02, to RMW.
- NIH, U01 AI150739, to JEC.
- NIH, contract HHS 75N93019C00074, to JEC.
- NIH, R01 GM129325, to Resource for Biocomputing, Visualization, and Informatics, University of California, San Francisco.

## Supplementary Material

Supplemental data

Supporting data values

## Version 1 12/01/2025

Electronic publication
