# Supplementary material for: A protective and heterosubtypic antibody lineage targeting the influenza A virus neuraminidase active site
Source: J Clin Invest. 2025 Dec 1;135(23):e188887. doi: 10.1172/JCI188887 (PMC12646661; doi:10.1172/JCI188887)
Supplement: Supplemental data [file jci-135-188887-s278.pdf]

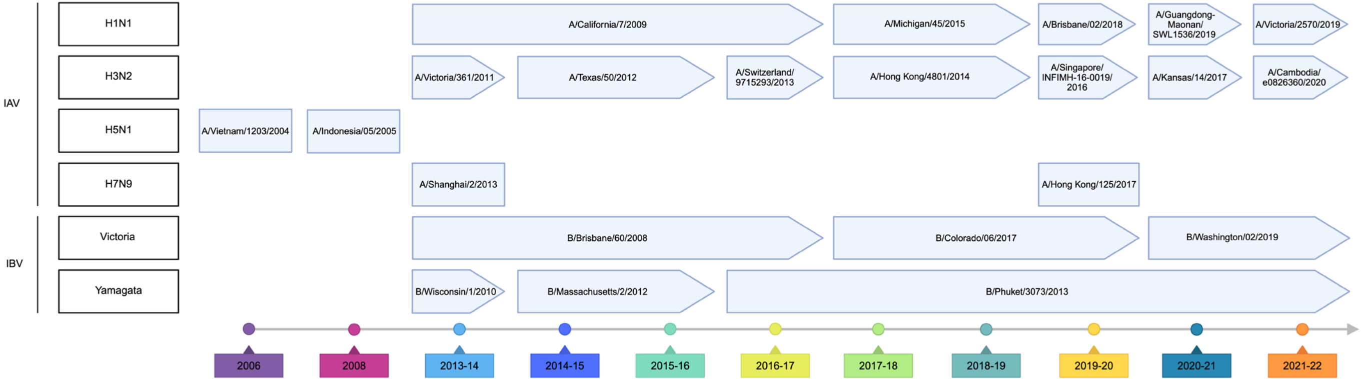

Supplemental Figure 1. Influenza vaccination schedule and formulations received by Donor 269. Timeline illustrating the seasonal influenza vaccinations recieved by Donor 269 from 2013 - 2021. Individual strains of the annual quadrivalent influenza vaccines are indicated for each year. Also shown are avian influenza strains (*i.e.*, H5N1 and H7N9) received by Donor 269 in the context of vaccine clinical trials. Created in BioRender. Sornberger, T. (2025) https://BioRender.com/cpa2mcz.

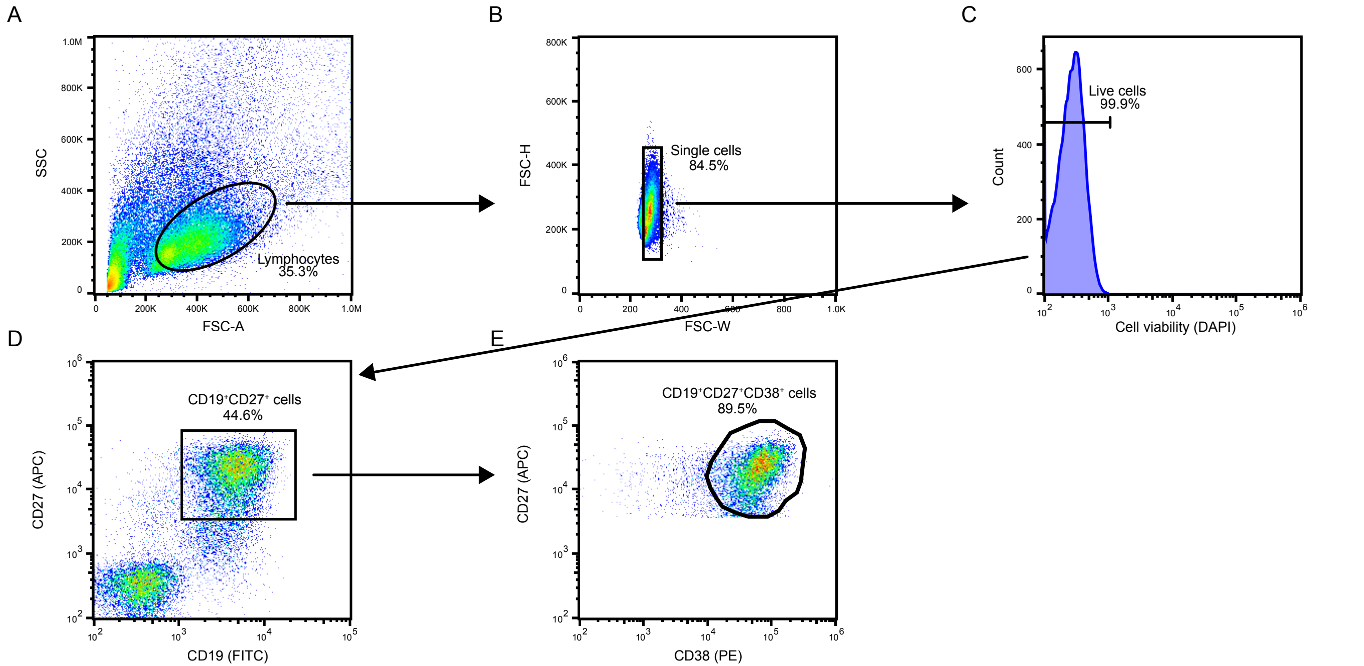

Supplemental Figure 2. Representative gating strategy for plasmablast isolation following 2017 H7N9 vaccination. Plasmablasts were enriched from PBMCs using a magnetic bead-based negative-selection kit and stained with phenotyping antibodies. **(A)** Lymphocytes were selected via the distribution of cells in the light scatter based on size (FSC) and intracellular composition (SSC). **(B)** Individual cells from the total B cell population were isolated based on cell width (FSC-W) and height (FSC-H). **(C)** DAPI staining was used to discriminate live/dead cells. Bar indicates the fraction of viable cells in the sample analyzed. **(D)** $CD19^{+}CD27^{+}$ B cells were identified using phenotyping antibodies anti-CD19-FITC and anti-CD27-APC. **(E)** $CD19^{+}CD27^{+}CD38^{+}$ B cells were identified from the $CD19^{+}CD27^{+}$ population using an anti-CD38-PE phenotyping antibody. Arrows indicate the gate from which each plot is derived. Numbers indicate the percentage of cells within each gate relative to the total number of cells.

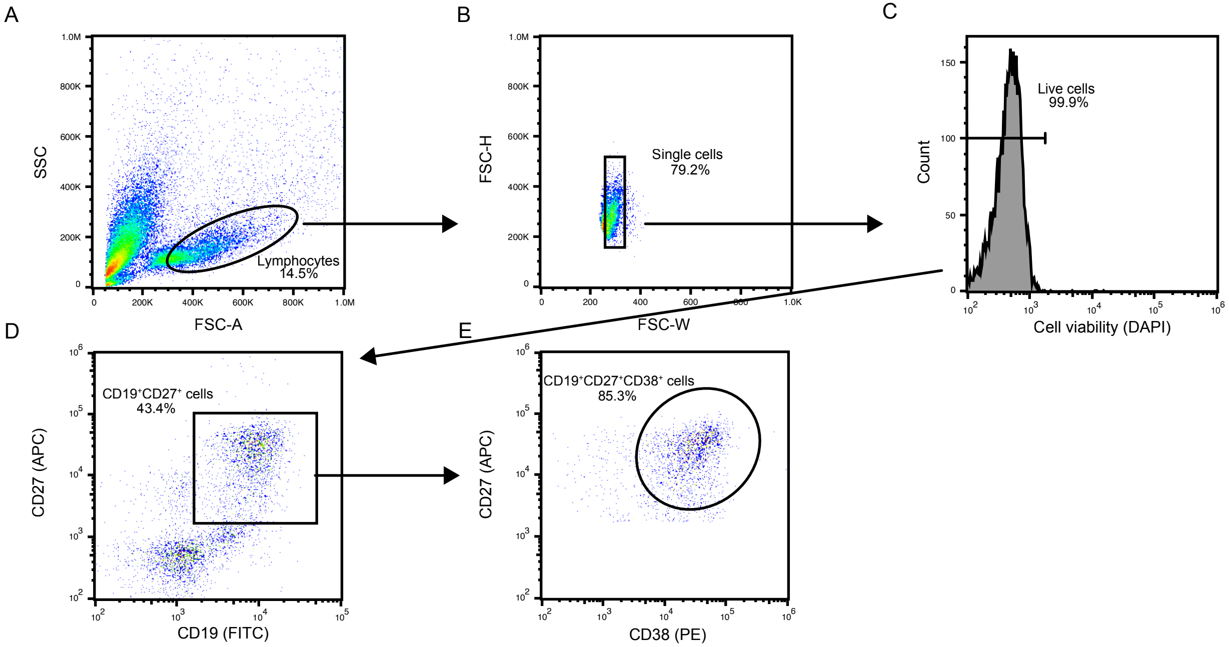

Supplemental Figure 3. Representative gating strategy for plasmablast isolation following 2020-2021 seasonal vaccination. Plasmablasts were enriched from PBMCs using a magnetic bead-based negative-selection kit and stained with phenotyping antibodies. **(A)** Lymphocytes were selected via the distribution of cells in the light scatter based on size (FSC) and intracellular composition (SSC). **(B)** Individual cells from the total B cell population were isolated based on cell width (FSC-W) and height (FSC-H). **(C)** DAPI staining was used to discriminate live/dead cells. Bar indicates the fraction of viable cells in the sample analyzed. **(D)** $CD19^{+}CD27^{+}$ B cells were identified using phenotyping antibodies anti-CD19-FITC and anti-CD27-APC. **(E)** $CD19^{+}CD27^{+}CD38^{+}$ B cells were identified from the $CD19^{+}CD27^{+}$ population using an anti-CD38-PE phenotyping antibody. Arrows indicate the gate from which each plot is derived. Numbers indicate the percentage of cells within each gate relative to the total number of cells.

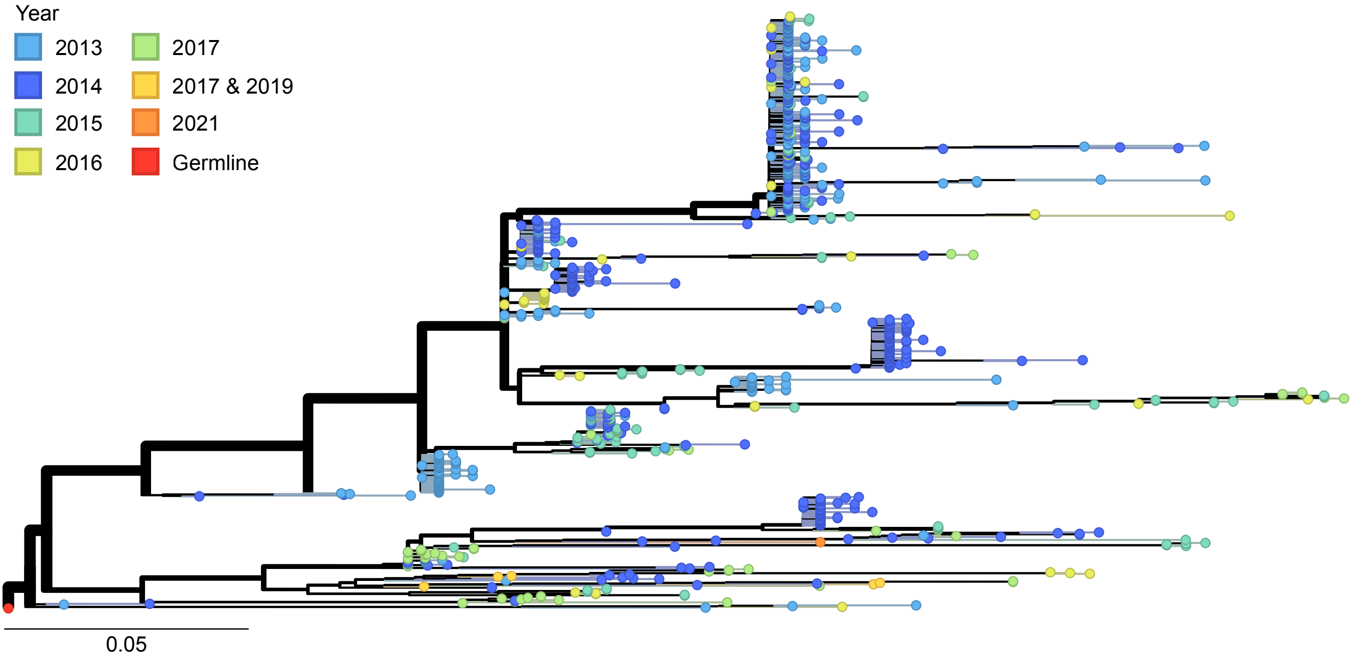

Supplemental Figure 4. Phylogeny of the entire FluA B cell lineage. Maximum-likelihood phylogenetic tree of unique heavy chain variable region sequences of the FluA B cell lineage generated using Augur and visualized using Auspice (89, 92). Full-length somatically mutated heavy chain sequences are rooted to the germline heavy chain variable gene (*IGHV1-18*04*). Tips indicate all related paired and unpaired heavy chain gene sequences. Colors indicate the year(s) in which each sequence was acquired and the rooted germline sequence. Scale bar denotes the average number of nucleotide substitutions per site.

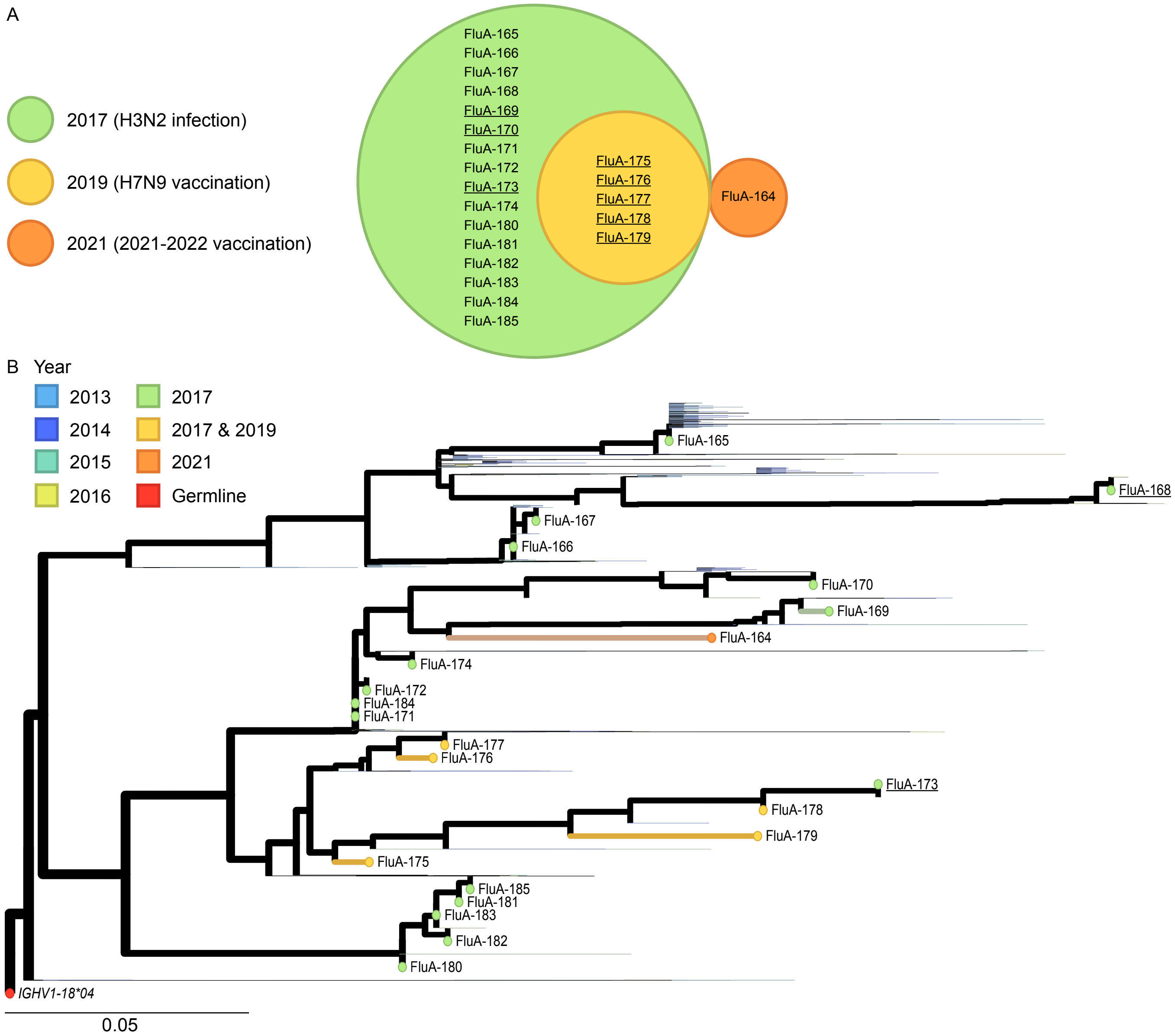

Supplemental Figure 5. Discovery and phylogeny of FluA mAbs expressed for binding reactivity. (A) Area-proportional Venn diagram depicting the overlap of each year(s) in which recombinantly expressed mAbs were identified. Antibodies with binding reactivity to the IAV N9 subtype are underlined. Figure generated using BioVenn (70). **(B)** Maximum-likelihood phylogenetic tree of heavy chain variable region sequences of the FluA B cell lineage generated using Augur and visualized using Auspice (89, 92). Somatically mutated heavy chain variable region sequences are rooted to the germline heavy chain variable gene (*IGHV1-18*04*). Tip labels indicate heavy chain gene sequences of recombinantly expressed mAbs tested for binding reactivity on ELISA. Colors indicate the year(s) in which each mAb sequence was acquired and the rooted germline sequence. Candidate mAbs FluA-168 and FluA-173 are underlined. Scale bar denotes the average number of nucleotide substitutions per site.

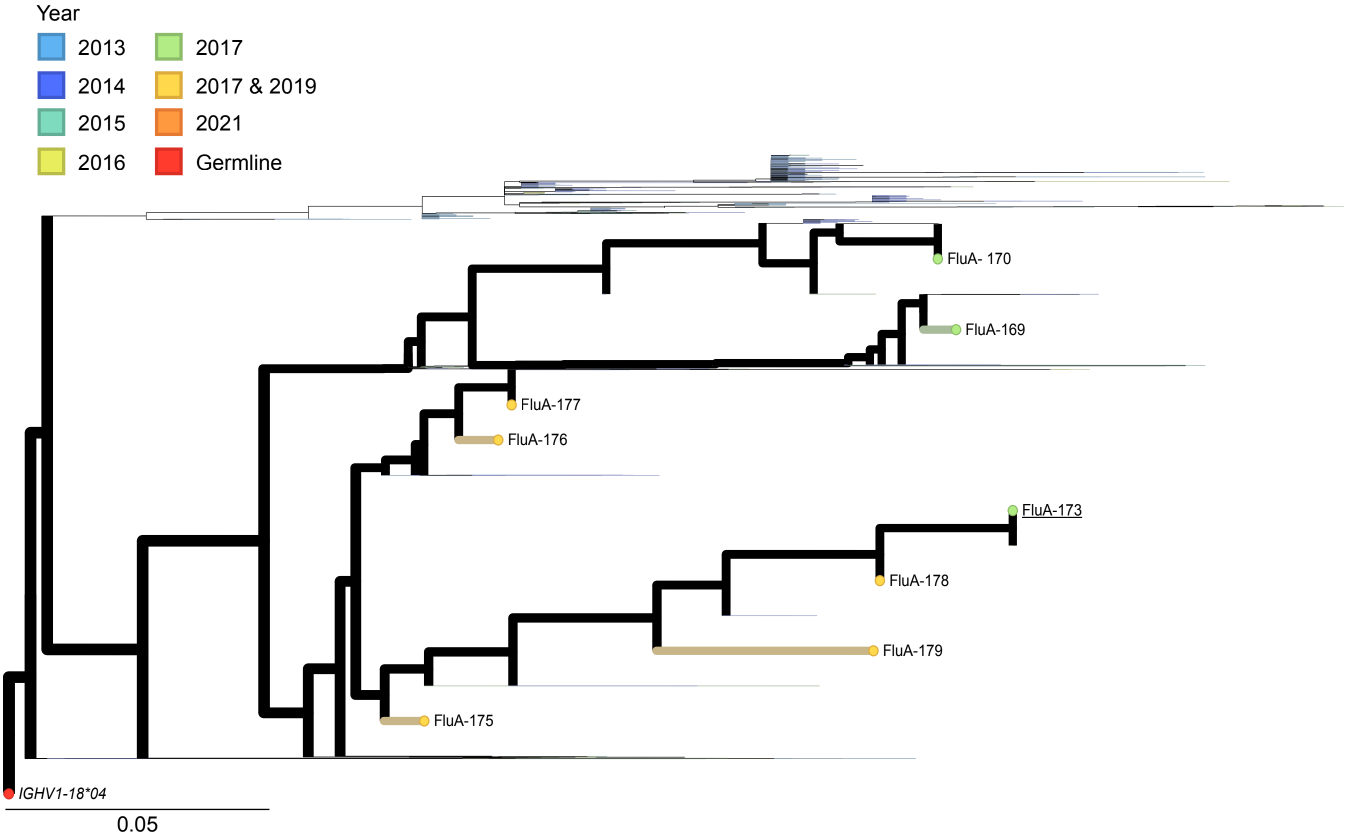

Supplemental Figure 6. Phylogeny of FluA mAbs reactive to the IAV N9 subtype. Maximum-likelihood phylogenetic tree of unique heavy chain variable region sequences of the FluA B cell lineage generated using Augur and visualized using Auspice (89, 92). Somatically mutated heavy chain variable region sequences are rooted to the germline heavy chain variable gene (*IGHV1-18*04*). Tip labels indicate heavy chain gene sequences of recombinantly expressed mAbs reactive to rN9 antigen on ELISA. Colors indicate the year(s) in which each mAb sequence was acquired and the rooted germline sequence. Candidate mAb FluA-173 is underlined.Scale bar denotes the average number of nucleotide substitutions per site.

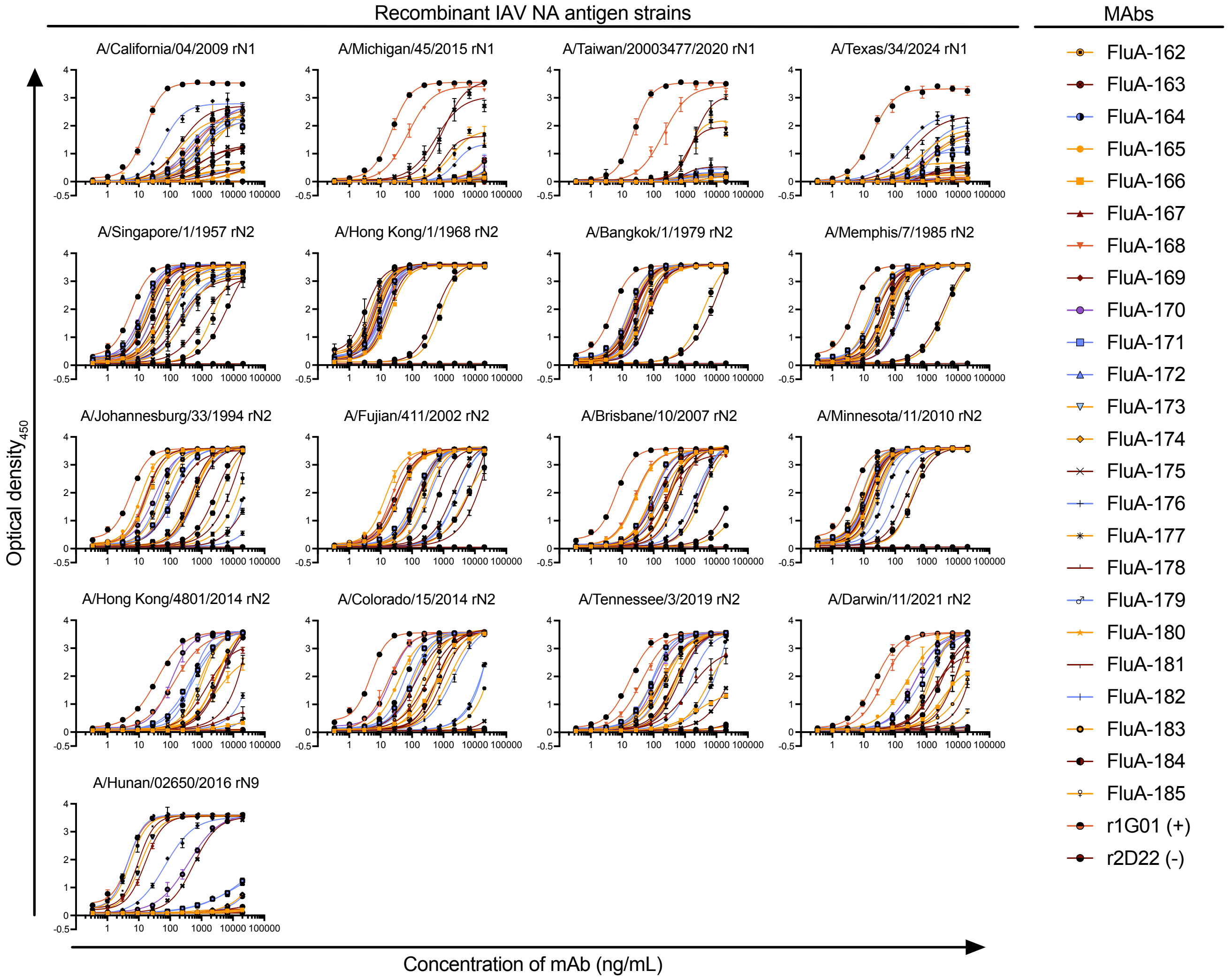

Supplemental Figure 7. Binding reactivity to recombinant NA antigen on ELISA. Dose-response curves of FluA and control mAbs binding to recombinant IAV NA antigens as indicated. The x-axis indicates the concentration of mAb (ng/mL) on a log-transformed scale, and the y-axis indicates the optical density of the reaction measured at 450 nm. Data are shown as mean ± SD. All mAbs were evaluated in technical triplicate across two biological replicates (shown is Replicate 1). Data for recombinant N1 antigens are reproduced from Figure 4A for sake of comparison.

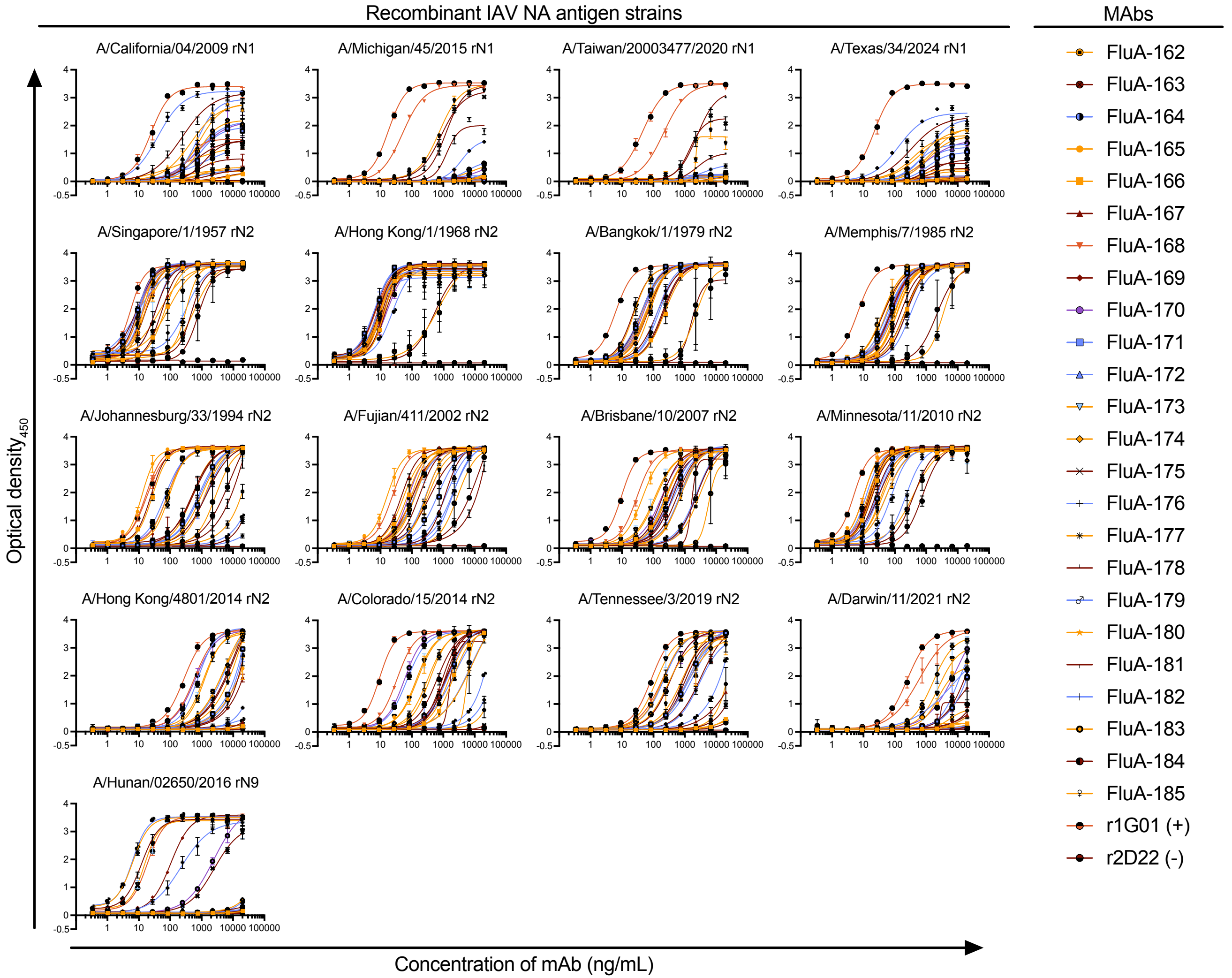

Supplemental Figure 8. Binding reactivity to recombinant NA antigen on ELISA. Dose-response curves of FluA and control mAbs binding to recombinant IAV NA antigens as indicated. The x-axis indicates the concentration of mAb (ng/mL) on a log-transformed scale, and the y-axis indicates the optical density of the reaction measured at 450 nm. Data are shown as mean ± SD. All mAbs were evaluated in technical triplicate across two biological replicates (shown is Replicate 2). Data for recombinant N1 antigens are reproduced from Figure 4A for sake of comparison.

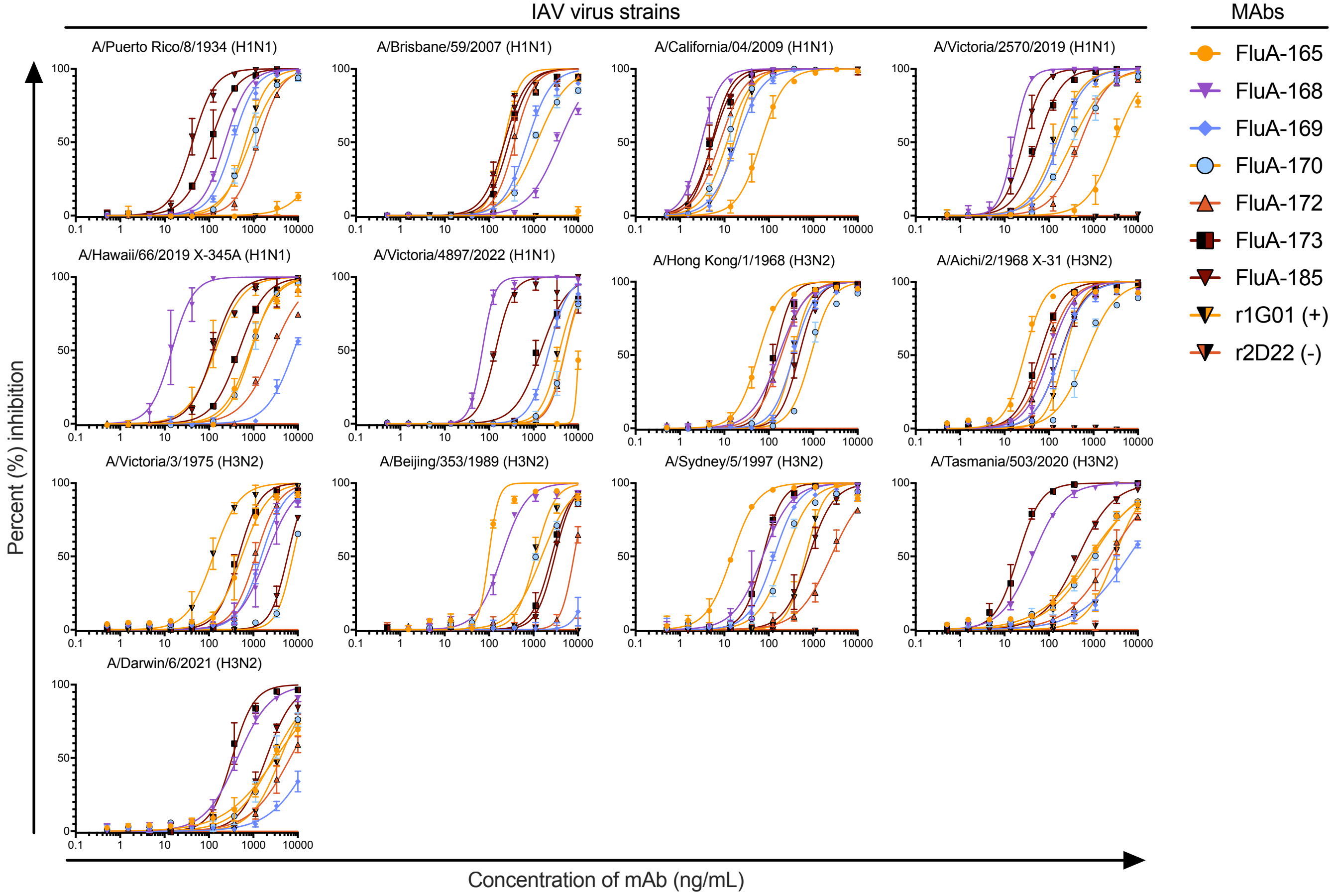

Supplemental Figure 9. Inhibition of sialidase activity by mAbs on ELLA. Dose-response curves of FluA and control mAbs inhibiting cleavage of terminal sialic acid residues from a fetuin glycoprotein by H1N1 and H3N2 virus strains as indicated. The x-axis indicates the concentration of mAb (ng/mL) on a log-transformed scale, and the y-axis indicates the percent (%) inhibition of sialidase activity. Data are shown as mean ± SD. All mAbs were evaluated in technical triplicate across two biological replicates (shown is Replicate 1).

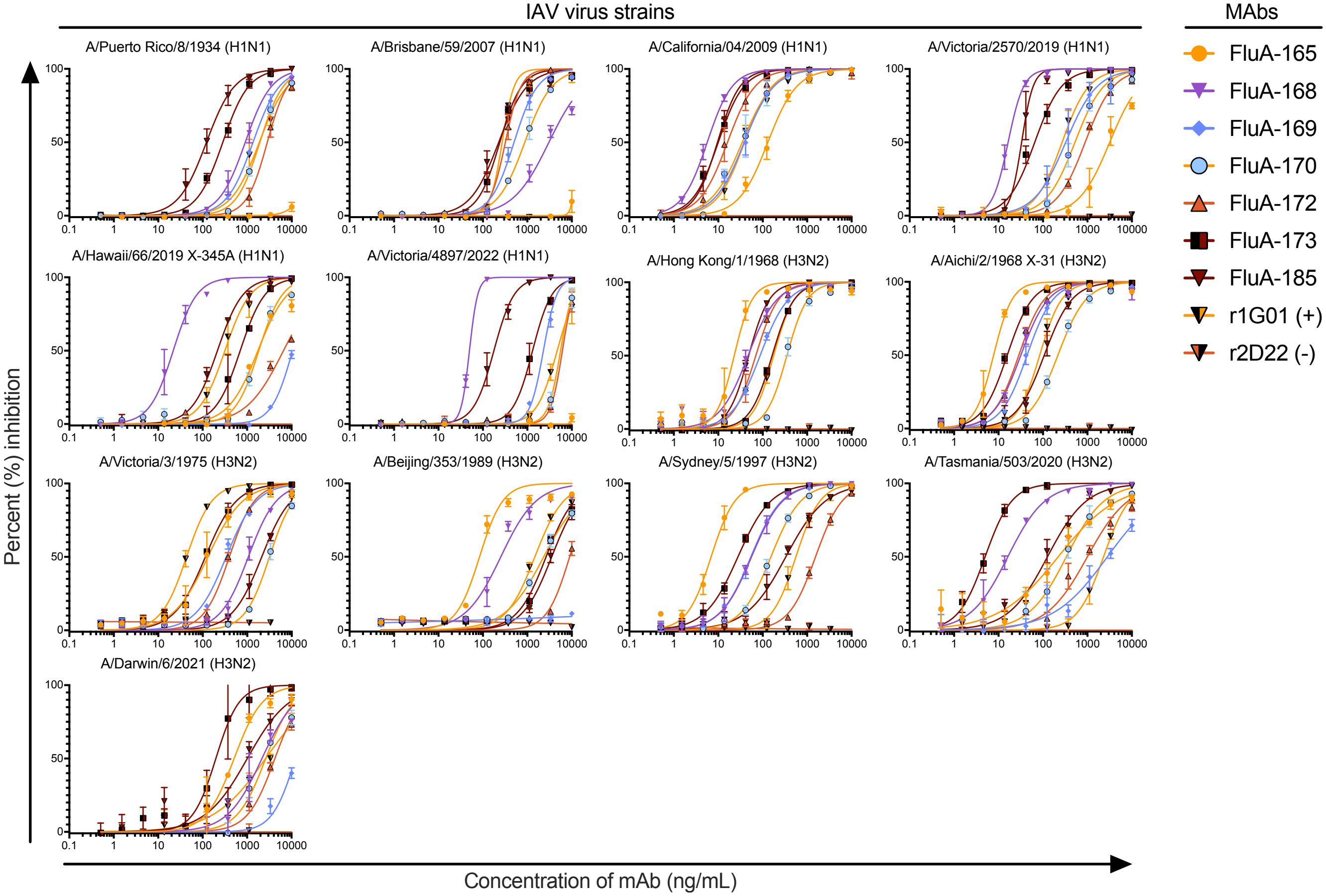

Supplemental Figure 10. Inhibition of sialidase activity by mAbs on ELLA. Dose-response curves of FluA and control mAbs inhibiting cleavage of terminal sialic acid residues from a fetuin glycoprotein by H1N1 and H3N2 virus strains as indicated. The x-axis indicates the concentration of mAb (ng/mL) on a log-transformed scale, and the y-axis indicates the percent (%) inhibition of sialidase activity. Data are shown as mean ± SD. All mAbs were evaluated in technical triplicate across two biological replicates (shown is Replicate 2).

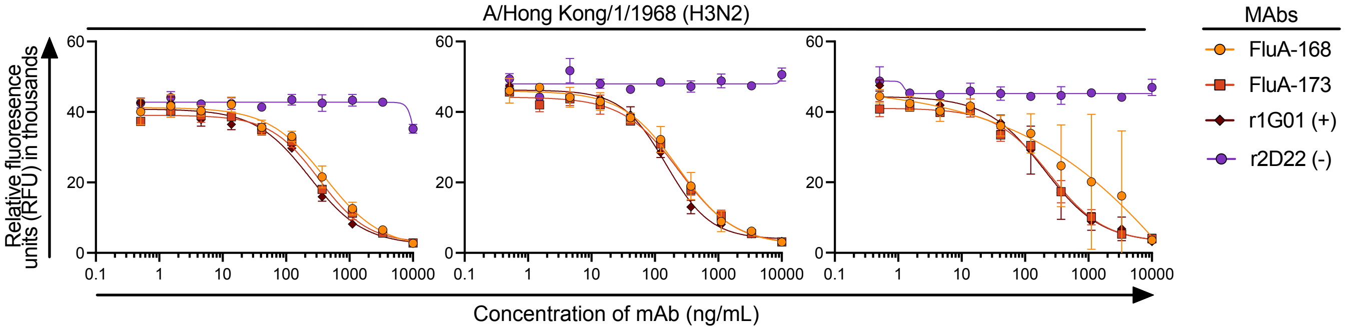

Supplemental Figure 11. Inhibition of sialidase activity by mAbs on the NA-Fluor™ assay. Dose-response curves of FluA and control mAbs inhibiting cleavage of a fluorescent MUNANA substrate by an H3N2 virus (A/Hong Kong/1/1968). The x-axis indicates the concentration of mAb (ng/mL) on a log-transformed scale, and the y-axis indicates the relative fluorence units (RFU) in thousands. Data are shown as mean ± SD. All mAbs were evaluated in technical triplicate across three biological replicates (shown Left-to-Right are Replicates 1-3). Data for Replicate 2 are reproduced from Figure 2B for sake of comparison.
